# The draft genome assembly of *Rhododendron delavayi* Franch. var. *delavayi*

**DOI:** 10.1093/gigascience/gix076

**Published:** 2017-08-26

**Authors:** Lu Zhang, Pengwei Xu, Yanfei Cai, Lulin Ma, Shifeng Li, Shufa Li, Weijia Xie, Jie Song, Lvchun Peng, Huijun Yan, Ling Zou, Yongpeng Ma, Chengjun Zhang, Qiang Gao, Jihua Wang

**Affiliations:** 1 Flower Research Institute of Yunnan Academy of Agricultural Sciences, National Engineering Research Center For Ornamental Horticulture, No. 2238 Beijing Road, Panlong District, Kunming 650205, China; 2 Key Lab of Yunnan Flower Breeding, No. 2238 Beijing Road, Panlong District, Kunming 650205, China; 3 BGI-Shenzhen, BGI Park, No. 21 Hongan 3rd Street, Yantian District, Shenzhen 518083, China; 4 Kunming Botanical Garden, Kunming Institute of Botany, Chinese Academy of Sciences, No. 132 Lanhei Road, Panlong District, Kunming, Yunnan 650201, China; 5 Germplasm Bank of Wild species, Kunming Institute of Botany, Chinese Academy of Sciences, No. 132 Lanhei Road, Panlong District, Kunming, Yunnan 650201, China

**Keywords:** *Rhododendron delavayi*, genomics, genome assembly, annotation

## Abstract

*Rhododendron delavayi* Franch. is globally famous as an ornamental plant. Its distribution in southwest China covers several different habitats and environments. However, not much research had been conducted on *Rhododendron* spp. at the molecular level, which hinders understanding of its evolution, speciation, and synthesis of secondary metabolites, as well as its wide adaptability to different environments. Here, we report the genome assembly and gene annotation of *R. delavayi* var. *delavayi* (the second genome sequenced in the Ericaceae), which will facilitate the study of the family. The genome assembly will have further applications in genome-assisted cultivar breeding. The final size of the assembled *R. delavayi* var. *delavayi* genome (695.09 Mb) was close to the 697.94 Mb, estimated by k-mer analysis. A total of 336.83 gigabases (Gb) of raw Illumina HiSeq 2000 reads were generated from 9 libraries (with insert sizes ranging from 170 bp to 40 kb), achieving a raw sequencing depth of ×482.6. After quality filtering, 246.06 Gb of clean reads were obtained, giving ×352.55 coverage depth. Assembly using Platanus gave a total scaffold length of 695.09 Mb, with a contig N50 of 61.8 kb and a scaffold N50 of 637.83 kb. Gene prediction resulted in the annotation of 32 938 protein-coding genes. The genome completeness was evaluated by CEGMA and BUSCO and reached 95.97% and 92.8%, respectively. The gene annotation completeness was also evaluated by CEGMA and BUSCO and reached 97.01% and 87.4%, respectively. Genome annotation revealed that 51.77% of the *R. delavayi* genome is composed of transposable elements, and 37.48% of long terminal repeat elements (LTRs). The *de novo* assembled genome of *R. delavayi* var. *delavayi* (hereinafter referred to as *R. delavayi*) is the second genomic resource of the family *Ericaceae* and will provide a valuable resource for research on future comparative genomic studies in *Rhododendron* species. The availability of the *R. delavayi* genome sequence will hopefully provide a tool for scientists to tackle open questions regarding molecular mechanisms underlying environmental interactions in the genus *Rhododendron*, more accurately understand the evolutionary processes and systematics of the genus, facilitate the identification of genes encoding pharmaceutically important compounds, and accelerate molecular breeding to release elite varieties.

## Background

*Rhododendron* L. is a genus in the family Ericaceae. It is 1 of the largest and most diverse genera in the family and is distributed predominantly throughout the Northern hemisphere, but also reaches into the Asian tropics. More than 1000 species of *Rhododendron* are currently recognized, of which 567 species representing 6 subgenera are known from China. Of these Chinese species, approximately 80% are endemic [1, 2]. Because of the adaptability of this genus to different environments, species such as *R. arboreum* and *R. ferrugineum* have been used to investigate the effects of different environmental factors on plant growth, development, and domestication [3–6].

Certain secondary metabolites in *Rhododendron* have been investigated in connection with antioxidant, anti-inflammatory, anti-carcinogen, and anti-bacterial properties; these compounds have potential in the alleviation of symptoms in conditions including diabetes, arthritis, headache, and hypertension [7–9]. Genome-level sequencing could help investigation into genes responsible for these metabolites and could facilitate the characterization of bio-active compounds and downstream production.

Most species of *Rhododendron* are diploid (2*n* = 26). The relatively low levels of ploidy and reported introgression of genetic material between species in nature might be important in the evolution and speciation of *Rhododendron* [10]. Hybrid varieties can be produced with relative ease by using *Rhododendron* as the parent because of its natural interspecific hybridization [11, 12]. Previous research on morphology, anatomy, and cytology of *Rhododendron* suggests that the subgenus *Hymenanthes* represents a basal state of this genus [13], but classification attempts that employed only a small set of gene regions were not able to resolve relationships within the subgenus [14, 15].

**Figure 1:**
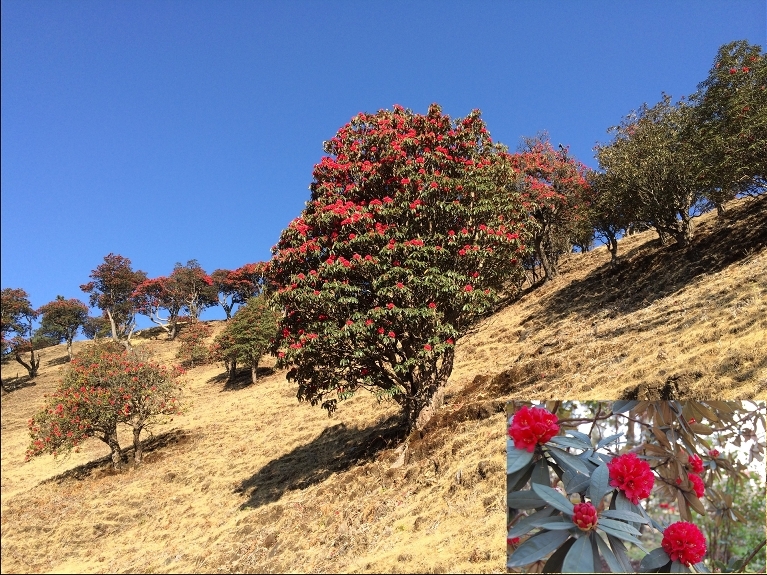
*Rhododendron delavayi* Franch. var. *delavayi* on Cang Shan Mountain, Dali.

*R. delavayi* Franch. is widely distributed throughout southwest China and grows at a wide altitudinal range, between 1200 and 3200 m. The species belongs to the subgenus *Hymenanthes*, subsection *Arborea* [1, 16]. Four varieties have been described for this species. *Rhododendron delavayi* var. *peramoenum* has narrow leaves and has been reported in western Yunnan, northeast India, and Myanmar, whereas *R. delavayi* var. *delavayi* has broader leaves than the former and mainly dominates in the Chinese range of the species. Another 2 varieties, *R. delavayi* var. *adenostylum* and *R. delavayi* var. *pilostylum*, were recently shown to fall within the spectrum of morphologies observed in hybrids between *R. delavayi* and *R. irroratum* [17]. In this project, material obtained from *R. delavayi* var. *delavayi* (see as Fig. 1) was used to generate genome sequences.

Due to its very attractive flowers and good resistance to arid and cold climates, *R. delavayi* has become a highly profitable ornamental flower in the market, especially in China and some Southeastern Asian countries, such as Vietnam, Thailand, Burma, and India. Nevertheless, it was believed that the anthropogenic activities have significantly reduced the diversity of plants of this genus in nature [18].

The aim of this project was to obtain a genome sequence of *R. delavayi*. With an available genome sequence, several next-generation sequencing approaches requiring a reference will become feasible, which will enable more in-depth research into genome-environment interactions, help with marker development for phylogenetic studies, and open possibilities for genome-assisted cultivar breeding and other downstream applications.

## Data Description

### Sample collection

Tissue samples were obtained from a 50-year-old tree growing in Jindian National Forest Park (Kunming, Yunnan, Taxonomy ID: 321363). This tree was transplanted from Cang Shan Mountain (Dali, Yunnan) in 1995. For genome library preparation, only leaf tissue was used; for transcriptome sequencing, samples were obtained from 5 different tissues: flowers, flower buds, young leaves, mature leaves, and young stems. After collection, tissues were immediately transferred into liquid nitrogen and stored until DNA and RNA extraction.

### Illumina sequencing strategy

Genomic DNA was extracted from the leaf tissue using a standard CTAB extraction [19]. Different methods were used to construct different insert size libraries. For the small insert libraries (170, 250, 500, and 800 bp), Illumina's protocols were used as follows (Illumina, San Diego, CA, USA): (i) genomic DNA was fragmented by nebulization with compressed nitrogen gas; (ii) DNA ends were polished, and an adenine was added to the ends of the fragments; (iii) DNA adaptors (Illumina) with a single “T” overhang at the 3’ end were ligated to the DNA fragments above; (iv) the ligation products were run on 2% agarose gels, and the bands corresponding to each insert size were excised. For the large insert libraries (2, 5, 10, 20, and 40 kb), Illumina's mate pair library protocols were followed: (i) genomic DNA was fragmented by nebulization with compressed nitrogen gas; (ii) DNA ends were polished using dNTPs labeled with biotin and circularized for self-ligation; (iii) circularized DNA was fragmented again by DNA Exonuclease, followed by enrichment of fragments containing biotin/streptavidin with magnetic beads; 4) fragment ends were further polished, followed by addition of an “A” base and adaptors to form the large insert libraries.

As shown in Table 1, the read length of the large insert libraries (2, 5, 10, 20, and 40 kb) was 49 bp, and the read length of the small insert libraries (170, 500, and 800 bp) was 100 bp, with the exception of the 250 bp insert library, which had a read length of 150 bp. A total of 336.83 Gb (×482.61) raw reads were generated from all constructed libraries. Before assembly, reads with low-quality polymerase chain reaction duplication and adapter contaminations were filtered by SOAPfilter, as included in SOAPdenovo v. 2.04 (SOAPdenovo2, RRID:SCR_014986) [20], and finally 246.06 Gb (×352.55) high-quality sequences were obtained for genome assembly.

**Table 1:** Sequencing libraries and data yields for whole-genome shotgun sequencing

Library type	Lane	Read length (bp)	Insert size (bp)	Raw bases		Clean bases	
				Total bases (Gb)	Depth (×)	Total bases (Gb)	Depth (×)
PE101	2	100	170	80.47	115.30	74.12	106.20
PE151	1	150	250	59.69	85.52	47.20	67.63
PE101	4	100	500	47.89	68.62	43.58	62.44
PE101	3	100	800	42.22	60.49	36.79	52.71
MP50	2	49	2000	30.36	43.50	19.56	28.03
MP50	3	49	5000	23.11	33.11	9.06	12.98
MP50	3	49	10 000	20.17	28.90	6.71	9.61
MP50	2	49	20 000	19.01	27.24	4.35	6.23
MP50	1	49	40 000	13.91	19.93	4.69	6.72
Total	21			336.83	482.61	246.06	352.55

Sequencing depth was calculated based on a genome size of 697.94 Mb. High-quality data were obtained by filtering raw data for low-quality and duplicate reads.

RNA of each tissue was extracted separately according to the TRIzol protocol (Invitrogen) and then combined in homogenized RNA concentration. Total mRNAs were purified from total RNA by Dynal Oilgo (dT) beads (Invitrogen). Random oligo-nucleotides and M-MuLV Reverse Transcriptase (RNase H) were used to synthesize the first cDNA strand, and then the second cDNA strand was synthesized using DNA Polymerase I and RNase H. The cDNA libraries with insert sizes of 200–500 base pairs (bps) were selected and purified with the AMPure XP beads system (Beckman Coulter), and subsequently sequenced on an Illumina HiSeq 2000 platform. Both cDNA library construction and Illumina sequencing were carried out by BGI-ShenZhen. Paired-end reads were generated with a read length of 90 bp. The raw reads were filtered by SOAPnuke (SOAPnuke, RRID:SCR_015025) [21], with the following criteria for being discarded: (i) reads contained adaptors; (ii) reads with unknown nucleotides larger than 5%; (iii) low-quality reads (the rate of reads at which quality value ≤ 10 is more than 20%). After filtering, 7.13 G clean reads were obtained for genome evaluation and gene annotation. All clean reads were uploaded to NCBI (SRA505613).

### Genome size estimates

We characterized the genome sequence (genome size, heterozygosity, and repetitive content) using the distribution of k-mers of 17, 21, 25, and 27 lengths from the clean reads (29 Gb clean reads from 500 and 800 bp insert size libraries). This analysis was performed using KmerFreq (included in SOAPdenovo, v. 2.04). The genome size (G) of *R. delavayi* was estimated by the following formula: G = *k*-mer_number/*k*-mer_depth, where the *k*-mer_number is the total number of *k*-mers, and *k*-mer_depth refers to the most frequent peak.

**Figure 2:**
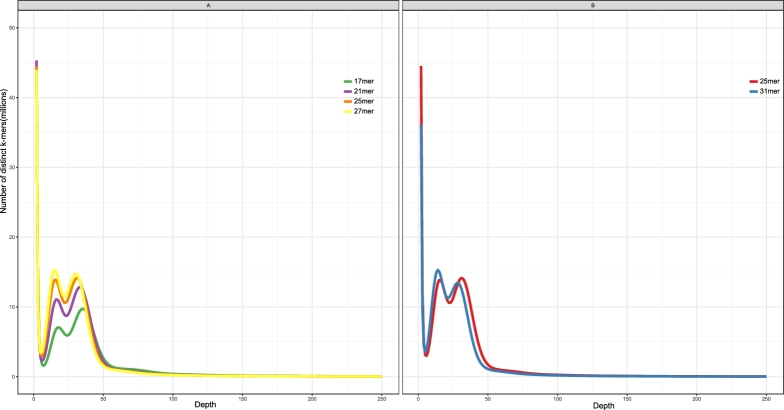
*k*-mer analysis of the *R. delavayi* genome. (**A**) Histograms of *k*-mer frequencies in the clean read data for *k* = 17 (green), *k* = 21 (purple), *k* = 25 (orange), and *k* = 27 (yellow) by KmerFreq. (**B**) Histograms of *k*-mer frequencies in clean data for *k* = 25 (red) and *k* = 31 (blue) by jellyfish. The x-axis shows the number of times a *k*-mer occurred; e.g., the peaks near x = 31 indicate the number of *k*-mers that occurred 31 times in the data.

All 4 *k*-mer distribution curves displayed 4 distinct peaks (Fig. 2A). The first peak at *k* = 1 was an artifact caused by sequencing errors, each of which created a *k*-mer that never occurred in the genome. The remaining 3 peak distributions indicated that the genome is a slightly repetitive, heterozygous, diploid genome. The third peak was a “diploid” peak (*k*-mers shared between homologous chromosomes) and was twice as deep as the second “haploid” peak (*k*-mers unique to a haplotype due to heterozygosity). The fourth peak was a repetitive peak (k-mers duplicated due to repetition) and was twice as deep as the “diploid” peak. For *k* = 17, the homozygous peak (the third peak) was found at a depth of ∼×35, with a k-mer_number of 24 427 946 424 and k-mer_depth of 35. The *R. delavayi* genome size was estimated to be 695.94 Mb, and the data used in 17-mer analysis was about ×41.7 coverage of the genome. All the k-mer sizes yielded similar genome size estimates of ∼697–717 Mb (Table 2).

**Table 2:** Statistics of genome size estimation by KmerFreq with *k* = 17, 21, 25, and 27

Genome	K-mer length (bp)	K-mer numbers	K-mer depths	Estimated genome size	Read numbers	Genome coverage
*R. delavayi*	17	24 427 946 424	35	697 941 326	290 808 886	×41.8
	21	23 264 710 880	33	704 991 238	290 808 886	×41.25
	25	22 101 475 336	31	712 950 817	290 808 886	×40.79
	27	21 519 857 564	30	717 328 585	290 808 886	×40.54

The genome size was estimated according to the formula Genome size = *k*-mer_numbers/*k*-mer_depths.

We also used jellyfish v. 2.0 (jellyfish, RRID:SCR_005491) [22] to make *k*-mer histograms for k-mers 25 and 31 (Fig. 2B), and genome size estimates were 693 and 703 Mb, respectively (Table 3). The *k*-mer distribution obtained by jellyfish showed a similar trend to KmerFreq. Using the result from jellyfish as input for GenomeScope [23], heterozygosity estimates for the *R. delavayi* genome were in the range of ∼0.9–1.1%.

**Table 3:** Properties of the *R. delavayi k*-mer distributions for *k* = 25 and *k* =31 using jellyfish

k-mer length	*k* = 25	*k* = 31
Total *k*-mers	22 120 556 922	20 373 342 031
Error *k*-mers	615 612 427	688 273 368
Haploid coverage depth	16	14
Diploid coverage depth	31	28
Diploid genome size	693 707 887	703 038 167

The genome size was estimated according to the formula Genome size = (Total *k*-mers—Error *k*-mers)/Diploid coverage depth.

### Genome and transcriptome assembly

The *Rhododendron delavayi* genome was assembled using Platanus v. 1.2.4 (Platanus, RRID:SCR_015531) [20], employing the 3 following steps: contig assembly, scaffolding, and gap closing. For the contig assembly step, the command line parameters “platanus assemble -t 20 -m 300 -u 0.2 -d 0.5 -k 41 -s 10” were specified to construct de Bruijn graphs for small insert size libraries (170, 250, 500, and 800 bp), to modify the graphs, and to display the output sequences. With these options, Platanus increased the *k*-mer size by the step size *k*_step_ (default 10) and iteratively reconstructed the graphs. Assembled contigs and bubbles in the graphs were obtained from this step. In the scaffolding step, the bubbles and reads from the libraries with small insert sizes (170, 250, 500, and 800 bp) and large insert sizes (2, 5, 10, 20, and 40 kb) were mapped onto the assembled contigs for scaffold construction. The command used for this was “platanus scaffold -t 20 -u 0.2 -c contigs.fasta -b bubble.fasta -IP <reads from small insert size libraries>” -OP <reads from large insert size libraries.> In the gap-filling step, the command used was “platanus gap_close –t 20 –IP <reads from small insert size libraries,>” and gaps within scaffolds were filled by reads from small insert size libraries where 1 end could be mapped to 1 contig and the other end extended into a gap. Two more gap-filling steps were performed based on the assembly results, first utilizing KGF (v. 1.06) [24], followed by GapCloser v. 1.12-r6 (GapCloser, RRID:SCR_015026) [24].

To remove a probable redundant sequence in the genome, we used jellyfish v. 2.0 to calculate the 17-mer frequency table from all short insert libraries, then passed the result to trimDup, which comes as part of Rabbit (the software is also archived in the *Gigascience* repository, *Giga*DB) [25, 26, 27]. The following command was used “trimDup 17-mer_table 17 1.5*main_peak genome.fa 0.3.” Hence, *k*-mers were excluded if their frequency was higher than 1.5 times the main peak. Each *k*-mer was defined as either a “repeat” or a “unique” *k*-mer, depending on whether its occurrence frequency was greater or less than twice the average frequency. Rabbit uses a Poisson-based *k*-mer model to establish a 17-mer frequency table from each scaffold of the genome sequences and then determines unique *k*-mers belonging to each scaffold and common *k*-mers shared by the scaffolds. The 17-mer frequency table generated in jellyfish is then used to filter the scaffolds so that the ratio of common to unique *k*-mers reaches 0.3. After the removal of 57.52 Mb of redundant scaffolds, a total scaffold length of 695 Mb was generated (Table 4). The contig N50 was 61.81 Kb, and the scaffold N50 was 637.82 Kb, while scaffolds with lengths of less than 100 bp were excluded. Meanwhile, we also ran another *de novo* assembler, SOAPdenovo2 (SOAPdenovo2, RRID:SCR_014986), with various modifications of parameters, but the results (Table 5) from SOAPdenovo2 were not better than those generated above.

**Table 4:** The genome assembly and completeness of *R. delavayi*

	Contig		Scaffold	
	Size (bp)	Number	Size (bp)	Number
N50	61 801	2871	637 826	313
Minimum length	13		79	
Maximum length	581 429		3 407 404	
Total size	657 780 215		695 092 854	
Number (≥100 bp)		209 926		193 086
Number (≥2 kb)		20 175		4972
Number (≥100 kb)		1315		1230
Number (≥1 Mb)				140
CEGMA completeness				95.87% [238]
CEGMA partial				98.39% [244]
BUSCO completeness				92.8% [1337]
BUSCO fragment				1.8%% [26]

Numbers of genes that match CEGMA or BUSO are shown in square brackets.

**Table 5:** Statistics of the assembly with different parameters

Assembler	Assembly size (bp)	Contig N50 (bp)	Scaffold N50 (bp)	K-mer (bp)	Gapcloser	Rabbit
SOAP*denovo*2	854 390 781	900	3380	63	No	No
SOAP*denovo*2	543 175 156	1118	5946	37	No	No
SOAP*denovo*2	1 231 272 241	19 792	67 539	87	Yes	No
SOAP*denovo*2	796 221 798	25 301	104 917	87	Yes	Yes
Platanus	750 231 563	13 232	583 084	41	No	No
Platanus	809 870 271	7886	383 826	47	No	No
Platanus	752 607 346	54 782	584 190	41	Yes	No
Platanus	695 092 854	61 801	637 826	41	Yes	Yes

Transcript assembly was carried out in Trinity release-20130225 (Trinity, RRID:SCR_013048) [28] with the following parameters: minimum contig length 200 bp, min glue 3, group pair distance 280, path reinforcement distance 85, and min kmer covage 3. The TGI Clustering Tool (TGICL) v. 2.1 [29] was used to remove redundancies and merge the Unigenes with overlaps of at least 40 bp. Finally, a total of 83 515 Unigenes were obtained, with a mean length of 1014 bp and an N50 of 1727 bp.

### Genome evaluation

We evaluated the completeness of the genome assembly using Core Eukaryotic Genes Mapping Approach (CEGMA) v. 2.5 (CEGMA, RRID:SCR_015055) [30] and Benchmarking Universal Single-Copy Orthologs v. 2.0 (BUSCO, RRID:SCR_015008) [31], which assess genome completeness using the conserved genes from the NCBI Eukaryotic Clusters of Orthologous Groups (KOGs) and BUSCO databases, respectively. CEGMA results indicated that 95.97% of core eukaryotic genes were contained in our assembly (238 out of 248 core eukaryotic genes). BUSCO analysis resulted in 92.8% of plants set (embryophyta_odb9, downloaded from BUSCO) identified as complete (1337 out of 1440 BUSCOs). More detailed information is given in Table 4. The Unigenes were aligned to the *R. delavayi* genome using BLAT v. 0.36 (BLAT, RRID:SCR_011919) [32] with default parameters. The alignment indicated that the assembled genome of *R. delavayi* covered 96.98% of the Unigenes, 89.57% of the Unigenes with at least 90% coverage in 1 scaffold, and 98.90% of the Unigenes with at least 50% coverage in 1 scaffold, suggesting a high level of coverage (Table 6).

**Table 6:** The unigene coverage of transcriptome data by *R. delavayi* assembly

Data	Number	Total	Base coverage	>90% sequence in	>50% sequence in
set	unigenes	length (bp)	by assembly (%)	1 scaffold (%)	1 scaffold (%)
>200 bp	83 515	84 701 674	96.98	89.57	98.90
>500 bp	46 582	73 471 401	96.90	85.64	99.03
>1000 bp	29 816	61 377 043	96.80	82.85	99.08

### Repeat annotation

To identify tandem repeats, TRF v. 4.07 [33] was used with the following parameters: Match = 2, Mismatch = 7, Delta = 7, PM = 80, PI = 10, Minscore = 50, MaxPerid = 2000. In total 29 073 954 bp of tandem repeat sequences were detected, representing 4.18% of the *R. delavayi* genome. Transposable elements were identified by using homology and *de novo* methods. Homology: RepeatMasker v. 4.0.5 (RepeatMasker, RRID:SCR_012954) [34] was employed to identify transposable elements with RepBase library (version 20.04) [35], while RepeatProteinMask (v. 4.05) [36] was used to identify transposable elements against the TE protein database in RepBase. *De novo*: (i) RepeatModeler v. 1.07 (RepeatModeler, RRID:SCR_015027) [37] and LTR_FINDER v. 1.05 (LTR_Finder, RRID:SCR_015247) [38] were used to identify transposable elements; (ii) the results from RepeatModeler and LTR_FINDER were merged into a *de novo* repeat library; (iii) RepeatMasker was employed to categorize the genome sequence against the *de novo* repeat library. Finally, transposable elements identified by homology or *de novo* library within the same category were merged by overlap. Transposable elements accounted for 51.77% of the *R. delavayi* genome, while long terminal repeat elements (LTRs) represented the largest fraction (37.48%) of transposable elements (Table 7). The most abundant subtypes were *Copia* and *Gypsy*, representing 6.84% and 25.49% of the assembly genome, respectively.

**Table 7:** Transposable elements in the *R. delavayi* genome

	Repbase TE length	Protein TE length	*De novo* TE length	Combined TEs	
				Length	Percentage
DNA	7 882 501	7 328 645	69 812 249	77 776 557	11.19
LINE	4 811 976	12 454 813	31 065 638	36 834 088	5.30
SINE	125 792	0.00	869 547	991 785	0.14
LTR	34 884 681	52 469 776	257 040 066	260 532 496	37.48
Other	552	0.00	0.00	552	0.00
Unknown	0.00	0.00	4 565 754	4 565 754	0.67
Total	47 001 844	72 016 848	350 372 642	359 874 503	51.77

Repbase TEs means RepeatMask against Repbase; Protein TEs means RepeatProteinMask result against Repbase protein; *De novo* TEs means RepeatMask against the *de novo* library; Combined TEs means the combined result of the 3 steps.

### Gene prediction

**Figure 3:**
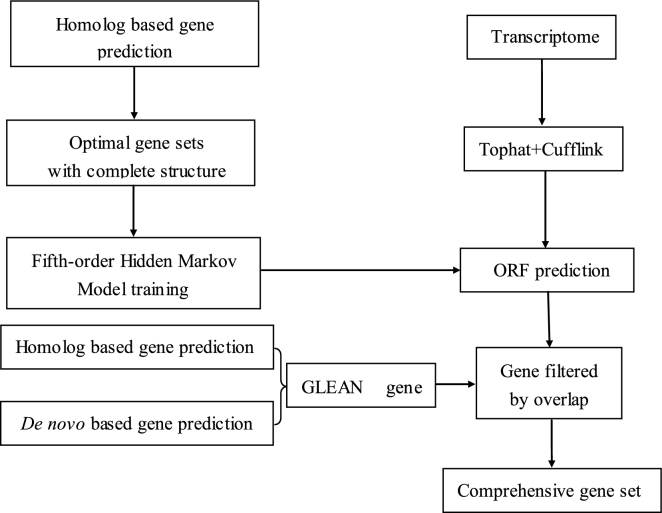
The gene prediction pipeline.

We combined homology-based, *de novo*, and transcript alignment methods to predict protein-coding genes in the *R. delavayi* genome. Four major steps were employed, and a detailed pipeline is given in Fig. 3.

For gene prediction based on homology, we obtained gene sets from *Arabidopsis thaliana* [37], *Actinidia chinensis* [39], *Capsicum annuum* [40], *Mimulus guttatus* [41], *Solanum tuberosum* [42], and *Solanum lycopersicum* [43]. For genes with alternative splicing variants, the longest transcript was selected to represent the gene. We aligned these homologous protein sequences to the *R. delavayi* genome using TBLASTN (v. 2.2.26) [44], employing an E-value threshold of 1e-5. The resulting BLAST hits were linked to candidate gene loci using solar (v. 0.9.6) [45] with options “-a prot2genome2 –z.” Then, we extracted the candidate gene locus sequences including 1 kb of flanking DNA upstream and downstream and used Genewise v. 2.2.0 (GeneWise, RRID:SCR_015054) [46] to define the intron-exon boundary. Genes with lengths of less than 150 bp or with erroneous structure (premature stop codon or frame shifts) were excluded from further analysis.

For the *de novo* prediction step, the repeat masked genome was used as input for 2 programs, AUGUSTUS v. 3.03 (Augustus: Gene Prediction, RRID:SCR_008417) [47] and GENSCAN v. 1.0 (GENSCAN, RRID:SCR_012902) [48]. To obtain a training set for AUGUSTUS, we randomly selected 5919 full-length genes that had been predicted based on homology, while for GENSCAN, Arabidopsis parameters were used. For the final non-redundant gene set, genes predicted based on both homology and *de novo* methods were combined with GLEAN (v. 1.0) [49], setting options “-gff -minlen 150 -minintron 11 -maxintron 15 000.” Genes with erroneous structure or short length were again excluded based on the same thresholds used for homology prediction.

For the transcript alignment prediction step, the short reads from the transcriptome data set generated in the previous step were mapped to the *R. delavayi* genome using Tophat v. 2.1.1 (TopHat, RRID:SCR_013035) [50] to identify the splice junctions. Cufflinks v. 2.2.1 (Cufflinks, RRID:SCR_014597) [51] was then used to assemble transcripts from the Tophat outputs. The coding potential of these transcripts was identified by using the same gene sets with a fifth-order Hidden Markov Model, which was achieved by the same gene sets used in the training of AUGUSTUS.

In the gene set combination step, outputs from GLEAN were combined with transcript assemblies as follows: first, translated sequences of both sets were cross-matched with an all-to-all BLASTP using an E-value cutoff of 1e-10. The matching transcript assemblies were then added to the GLEAN results as either untranslated regions (UTRs) or alternative splice forms, based on whether coverage and identity of the alignment results was larger than 0.9. The transcript assemblies that had no BLAST hit with the GLEAN results were added to the final set as novel genes.

As a result of these steps, a total of 32 938 non-redundant genes were predicted in the *R. delavayi* genome (Table 8). These genes were scattered over 2149 scaffolds, averaging 15.33 genes per scaffold.

**Table 8:** Summary of *R. delavayi* genome annotation

Gene		Gene numbers	Average gene	Average CDS	Average exons	Average exon	Average intron
set		of prediction	length (bp)	length (bp)	per gene	length (bp)	length (bp)
*De novo*	AUGUSTUS	42 672	2623.41	974.42	4.76	204.56	438.16
	GENSCAN	35 859	11 242.68	1186.91	6.35	186.87	1879.03
Homolog	*A. chinensis*	45 449	3501.48	846.20	3.21	263.43	1200.29
	*A. thaliana*	31 950	3724.50	994.90	4.07	244.30	888.41
	*C. annuum*	47 672	2558.30	805.26	3.01	267.50	872.00
	*M. guttatus*	34 616	3454.51	963.76	3.95	244.21	845.35
	*S. lycopersicum*	38 800	3324.95	917.11	3.74	245.47	880.01
	*S. tuberosum*	39 085	2958.21	850.18	3.22	263.79	948.30
	GLEAN	29 585	4126.65	1150.32	4.84	237.78	775.53
	RNA-seq	38 273	2989.97	828.78	3.45	240.07	881.29
	Final set	32 938	4434.22	1153.21	4.62	249.70	785.08

We also used Maker-P [52] to predict gene model with current homolog, *de novo*, and transcriptome results by using the parameter “est_gff, protein_gff, pred_gff” according to the Maker-P manual. The CEGMA assessment showed that our current pipeline identified 97.09% (234 of 241) of core eukaryotic genes, while the Maker-P pipeline identified only 86.72% (209 of 241) core eukaryotic genes. The BUSCO evaluation demonstrated that 87.4% and 6.4% of 1440 expected plant genes were identified as completeness and fragment, respectively (Table 9). Both assessment methods suggested that for the *R. delavayi* genome our current pipeline performed better than the Maker-P pipeline.

**Table 9:** BUSCO assessment of gene prediction with different pipelines

	Current pipeline		Maker-P	
BUSCO benchmark	Number	Percentage	Number	Percentage
Total BUSCO groups searched	1440		1440	
Complete single copy BUSCOs	1188	82.5	1056	73.3
Complete duplicated BUSCOs	70	4.9	67	4.7
Fragmented BUSCOs	92	6.4	152	10.6
Missing BUSCOs	90	6.2	165	11.4

### Functional annotation

Gene function annotation was assigned based on sequence and domain conservation. (i) Assignment based on sequence conservation: protein sequences of *R. delavayi* were aligned to KEGG (v. 76) [53] and SwissProt and TrEMBL (Uniprot release 201406) [54] by BLASTP (v. 2.2.26) using an E-value threshold of 1e-5. Best-hit BLAST results were then used to define the gene functions. (ii) Assignment based on domain conservation: InterProScan-5.11–51.0 (InterProScan, RRID:SCR_005829) [55] was employed to identify motifs and domains by matching against public databases Pfam [56], PRINTS [57], ProDom [58], SMART [59], and PANTHER [60]. Gene ontology identities [61] for each gene were then obtained from the corresponding InterPro entry [62]. Overall, 85.91% of genes were functionally annotated by at least 1 of the 5 databases above, with 22 946 InterPro entries, 16 471 GO entries, 21 210 KEGG entries, 22 693 SwissProt entries, and 27 975 TrEMBL entries (Table 10).

**Table 10:** Statistics for functional annotations in corresponding InterPRro entry

	Numbers of	Percent of
	matching genes	annotated genes
InterPro	22 946	69.66
GO	16 471	50.00
KEGG	21 210	64.39
Swissprot	22 693	68.90
TrEMBL	27 975	84.93
Annotated	28 296	85.91
Unannotated	4642	14.09

### Gene family construction

**Figure 4:**
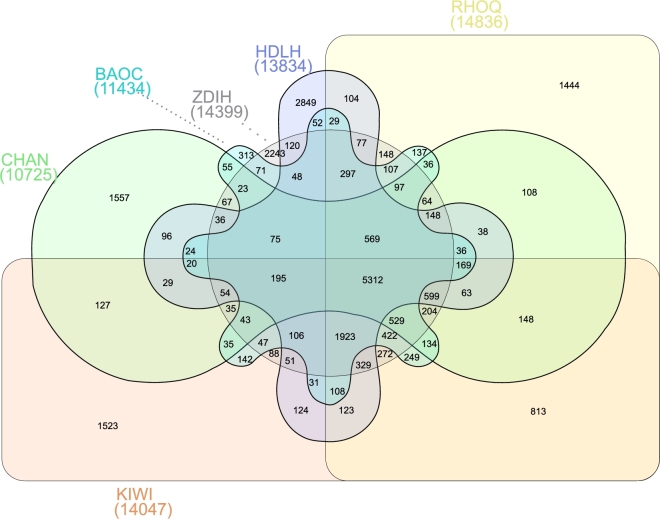
Groups of orthologues shared among the angiosperms *Rhododendron delavayi* (RHOQ), *Actinidia chinensis* (KIWI), *Primula veris* (BAOC), *Catharanthus roseus* (CHAN), *Phalaenopsis equestris* (HDLH), and *Tarenaya hassleriana* (ZDIH). Venn diagram generated by http://www.interactivenn.net/.

As references, protein sequences of 10 angiosperms (*Actinidia chinensis, Primula veris, Catharanthus roseus, Dendrobium officinale, Phalaenopsis equestris, Tarenaya hassleriana, Solanum tuberosum, Solanum lycopersicum, Arabidopsis thaliana*, and *Oryza sativa*) were downloaded (see the Supplementary Data). For genes with alternative splicing variants, the longest transcript was selected to represent the gene. Similarities between sequence pairs were calculated using BLASTP with an E-value threshold of 1e-5. Additionally, OrthoMCL (OrthoMCL DB: Ortholog Groups of Protein Sequences, RRID:SCR_007839) [63] was used with default parameters to identify gene family membership based on overall gene similarity combined with Markov Chain Clustering (MCL). Of all annotated genes, 77.60% were assigned to a family. A total of 14 836 families were represented, of which 1097 were specific of *Rhododendron delavayi* (Table 11). Figure 4 showed the number of orthologous gene families shared between 6 flower plant genomes, which have 5312 orthologous gene families in common with ancestral functions.

**Table 11:** The statistic results of gene family clusters

	Number of	Genes in	Unclustered	Number of	Unique	Average number of
Species	genes	families	genes	families	families	genes per family
*R. delavayi*	32 938	25 560	7378	14 836	1097	1.72
*A. chinensis*	39 040	26 061	12 979	14 047	1100	1.86
*P. veris*	18 269	15 080	3189	11 434	180	1.32
*C. roseus*	28 172	15 122	13 050	10 725	1231	1.41
*D. officinale*	35 474	25 525	9949	14 416	1091	1.77
*P. equestris*	29 413	21 086	8327	13 834	705	1.52
*T. hassleriana*	39 881	38 100	1781	14 399	623	2.65
*S. tuberosum*	34 879	28 093	6786	16 118	667	1.74
*S. lycopersicum*	33 585	25 623	7962	17 139	532	1.50
*A. thaliana*	26 637	23 007	3630	14 482	539	1.59
*O. sativa*	38 942	26 644	12 298	13 632	2020	1.95

### Phylogenetic analysis

For a phylogenetic analysis, 326 single copy orthologs were selected from the gene family step, and translated protein sequences were aligned in MUSCLE v. 3.8.31 (MUSCLE, RRID:SCR_011812) [64]. Next, the protein alignments were converted to corresponding coding sequences (CDS) using an in-house Perl script. Afterwards, the coding sequences of each single copy family were concatenated to form 1 supergene for each species. The nucleotides at positions 2 (phase 1 site) and 3 (4-fold degenerate site) of each codon were extracted separately and were used to construct 2 separate phylogenetic trees in PhyML3.0 (PhyML, RRID:SCR_014629) [65] specifying a HKY85 substitution model with a gamma distribution across sites. The tree using the phase 1 site was consistent with the tree using the 4 degenerate sites.

### Divergence time

**Figure 5:**
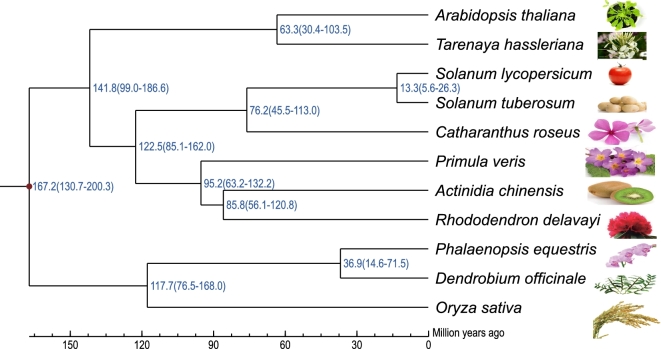
Estimation of divergence time. The blue numbers on the nodes are the divergence times from present; the red node indicates the calibrated split.

A Bayesian relaxed molecular clock approach was used to estimate species divergence time using MCMCTREE in PAML (PAML, RRID:SCR_014932) [66] based on the 4 degenerate sites data set used in phylogenetic analysis. When using previously published calibration times (split of *Oryza sativa* and *Arabidopsis thaliana* fixed as 130∼200 Mya) [67], the divergence time between *R. delavayi* and *Actinidia chinensis* was estimated to be in the range of 56.1–120.8 million years ago (Fig. 5).

## Conclusion

Now the order Ericales has 3 draft genome sequences of 3 economically important species (kiwi fruit [*Actinidia chinensis*], American cranberry [*Vaccinium macrocarpon*], and *R. delavayi*), 2 of which (*V. macrocarpon* and *R. delavayi*) also belong to the family Ericaceae. The availability of the *R. delavayi* genome sequence should facilitate *de novo* genome assembly of other species in this genus and, moreover, allow scientists to investigate interactions between environmental factors and related species at a molecular level. Furthermore, phylogenetic research can now draw on a genome as a resource to identify regions providing suitable resolution in this taxonomically difficult group, and it may become easier to identify the genes involved in metabolite pathways that have potential pharmaceutical importance.

## Availability of supporting data

Supporting data and the Rabbit software are available in the *Giga*DB database [27]. The raw data were deposited in the SRA527514 with project accession PRJNA361437 for the *Rhododendron delavayi* genome.

*Actinidia chinensis* (ftp://bioinfo.bti.cornell.edu/pub/kiwifruit/)

*Catharanthus roseus* (http://bioinformatics.psb.ugent.be/orcae/overview/Catro)

*Primula veris* (http://datadryad.org/resource/doi:10.5061/dryad.2s200)

*Dendrobium officinale* (ftp://202.203.187.112/genome/dendrobe/)

*Phalaenopsis equestris* (ftp://ftp.genomics.org.cn/from_BGISZ/20130120/)

*Solanum tuberosum*: phytozome12.0 (https://phytozome.jgi.doe.gov/pz/portal.html)

*Solanum lycopersicum*: phytozome12.0 (https://phytozome.jgi.doe.gov/pz/portal.html)

*Arabidopsis thaliana*: phytozome12.0 (https://phytozome.jgi.doe.gov/pz/portal.html)

*Oryza sativa*: phytozome12.0 (https://phytozome.jgi.doe.gov/pz/portal.html)

## Abbreviations

Gb: gigabase; GO: gene ontology; PE: paired-end; TE: transposable element.

## Competing interests

The authors declare that they have no competing interests.

## Funding

This project was supported by the Program of Science and Technology Talents Training in Yunnan province (2016HA005), the Program of Innovative Talents Promotion by the Chinese Ministry of Science and Technology (2014HE002), the Applied Basic Research Project of Yunnan Province (2016FB058), and the National Natural Science Foundation of China (31460217, 31560225).

## Author contributions

L.Z., J.W., Y.C., L.M., and Q.G. conceived the project. S.L., F.L., W.X., J.S., L.P., and H.Y. designed sample collection and extracted the genomic DNA. P.X. led the genome analysis, conducted the genome assembling, and predicted gene structure and repeat sequences. All of the authors listed above participated in discussions of the project and data. P.X., L.Z. (Lu Zhang), Q.G., and J.W. co-drafted the manuscript, and L.Z. (Ling Zou), Y.M., and C.Z. helped with manuscript revision. All authors read and approved the final manuscript.

## Supplementary Material

GIGA-D-17-00027_Original-Submission.pdf

GIGA-D-17-00027_Revision-1.pdf

GIGA-D-17-00027_Revision-2.pdf

Response-to-Reviewer-Comments_Original-Submission.pdf

Response-to-Reviewer-Comments_Revision-1.pdf

Reviewer-1-Report-(Original-Submission).pdf

Reviewer-2-Report-(Original-Submission).pdf

Reviewer-2-Report-(Revision-1).pdf

Reviewer-3-Report-(Original-Submission).pdf
