# Supplementary material for: The draft genome assembly of Rhododendron delavayi Franch. var. delavayi
Source: Gigascience. 2017 Aug 26;6(10):1–11. doi: 10.1093/gigascience/gix076 (PMC5632301; doi:10.1093/gigascience/gix076)

Manuscript Click here to download Manuscript 20170207Rhododendron delavayi.docx

The draft genome assembly of *Rhododendron delavayi* Franch.

Lu Zhang[1,2†], Pengwei Xu[3†], Yanfei Cai[1,2†], Lulin Ma[1,4†], Shifeng Li[1,2], Shufa Li[1,2], Weijia Xie[1,4], Jie Song[1,4], Lvchun Peng[1,2], Huijun Yan[1,2], Ling Zou[1,2], Chengjun Zhang[5], Qiang Gao[3*], Jihua Wang[1*]

Abstract

Rhododendron delavayi Franch., as one of the most representative species in Southwest of China, is a famous ornamental flower across the world. It adapts in different parts of Southwest plateau of China with different environmental factors. However, not much research had been conducted on *Rhododendron* sp. at molecular level, which hinders scientists to understand the nature of its wide adaptability to different environments, evolution, speciation and synthesis of pharmaceutically important compounds. Here, we report the first genome assembly of *R. delavayi*.

Findings: A total of 336.83 Gb of raw data were generated by the Illumina HiSeq 2000 platform, comprising approximately 483.26× coverage of the *R. delavayi* genome. After filtering out the low-quality data, PCR duplication and small insert size, 246.06 Gb of clean data was obtained. Assembling using clean reads by Platanus, a total scaffold length of 695 Mb was generated, with a scaffold N50 of 637 Kb. Gene annotation combined homolog, *de novo* and transcriptome assemblies, resulted in 32,938 protein-coding genes. The genome completeness was evaluated by CEGMA and BUSCO, reached 95.97 % and 92.8 % respectively. The Gene annotation completeness was also evaluated by CEGMA and BUSCO, reached 97.01 % and 87.4 %. Genome

*Correspondence: wjh0505@gmail.com; gaoqiang@genomics.cn
† Equal contributors
[1]The Flower Research Institute, Yunnan Academy of Agricultural Sciences, Kunming 650205, China
[2]National Engineering Research Center For Ornamental Horticulture, Kunming 650205, China
[3]BGI-Shenzhen, Shenzhen 518083, China
[4]Key Lab of Yunnan Flower Breeding, Kunming 650205, China
[5]Kunming Institute of Botany, Chinese Academy of Sciences, Kunming 650204, China

annotation reveals that 51.77 % of the *R. delavayi* genome is composed of transposable elements, and 37.48 % long terminal repeat elements (LTRs).

Conclusions: The *de novo* assembled genome of *R. delavayi* is the first genomic resource of the family *Ericaceae*, and will provide a valuable resource for research on future comparative genomic studies of *Rhododendron* species. The availability of *R. delavayi* genome sequence would enable scientists to more insightfully understand molecular mechanisms underlying interactions between environmental factors and *R. delavayi* plants, more accurately understand the evolution of *R. delavayi*, facilitate locating genes or loci accounted for pharmaceutically important compounds, and accelerate molecular breeding to release elite varieties.

Keywords: *Rhododendron delavayi*, Genomics, Assembly, Annotation

Background

In different parts of Sino-Himalayan region, the climates, topology, vegetation, altitudes and other environmental factors are highly different; over 1000 species of *Rhododendron* are widely distributed in this region [1]. Because of the adaptability of this genus to different environments, species such as *R. arboretum*, *R. ferrugineum* were used to investigate the effects of different environmental factors on plant growth, development, and domestication [2, 3, 4, 5].

Most species of *Rhododendron* are diploid (2n =2X= 26). The relatively small ploidy and reported transfer of genetic materials between species in nature might be important in evolution and speciation of *Rhododendron* [6], e.g. the natural interspecific hybridization is common between *R.delavayi* and *R.decorum* and *R.irroratum* [7, 8]. Methods to classify these species based on small set of primers often led to ambiguity in taxonomy [9]. Research on morphology, anatomy

and cytology of *Rhododendron* sp. suggested that the subgenus *Hymenanthes* apparently represents a primitive state of this genus; particularly, the subsect. *Fortunea*, *Auriculata*, *Grandia* and *Falconera* should be placed at the bottom of the evolution tree of genus *Rhododendron*, much research, particularly high-through-put sequencing, is needed to clarify the ambiguity [10].

Secondary metabolites are abundant in *Rhododendron* sp., such secondary metabolites could function as antioxidants, anti-inflammatory, anti-carcinogen, and anti-bacteria; these secondary metabolites might alleviate suffering from diabetes, arthritis, headache and hypertension [11, 12]. Sequencing at genome level and research on bioinformatics will help to investigate genes or loci accounted for these functions, and can facilitate characterizing bio-active compounds and down-stream production.

R. delavayi is widely distributed in southwest of China, belongs to subgenus *Hymenanthes*, section *Ponticum* and subsection *Arborea* [1, 13]. *R. delavayi* is a highly profitable ornamental flower in the market in China, because of its morphology is appealing to people in China and some Southeastern Asian countries. Anthropogenic activities have endangered this species, e.g. transformation of natural ecosystems to be used by humans, transplanting plants from natural habitats to gardens or as commodity in the market. It was believed that the anthropogenic activities have significantly reduced connectivity of plants of this genus in the nature [14]. *R. delavayi* is the most representative species of *Rhododendron* in Southwest of China, and widely adapted to arid and cold climates. In this project, we sequenced the genome of *Rhododendron delavayi* Franch. to facilitate research on effects of environmental factors on plants of *Rhododendron* at molecular level, taxonomy, phylogeny, molecular breeding and other down-stream applications.

Data description

Sample collection

The genomic DNA in this research was obtained from the leaf of a 50-year old plant (Fig.1; *Rhododendron delavayi* Franch.: Taxonomy ID: 321363), which was transplanted from Cang Shan Mountain of Dali to Jindian National Forest Park (Kunming, China) in 1995. For RNA sequence strategy, five different tissue types (flower, flower buds, young leaf, mature leaf and young stem) from this tree were sampled in order to obtain as many expressed genes as possible.

Illumina sequencing strategy

DNA of *Rhododendron delavayi* was extracted from the leaf tissue by a standard CTAB extraction [15]. Libraries with an insert size of 170, 250, 500 and 800 bp were prepared by following Illumina's protocols (San Diego, CA). For libraries with an insert size 2, 5, 10, 20 and 40 kb, such procedures as DNA circularization, digestion of linear DNA, fragmentation of circularized DNA and purification of biotinylated DNA were performed before adapter ligation. After library preparation and quality control of DNA samples, template DNA fragments were hybridized to the surface of flow cells on an Illumina Genome Analyzer II sequencer (GA2), amplified to form clusters, and sequenced following the standard Illumina manual. Finally, we generated 336.83 Gb of raw reads from all constructed libraries, raw sequenced outputs for each library were summarized in Table 1. Before assembly, reads with low quality, PCR duplication or adapter contamination were removed by using SOAP filter, a software application archived in SOAP *de novo* package [16]. After filtering, 246.06 Gb (353.03×) high-quality sequences were obtained for genome assembly.

RNA was extracted separately from each tissue according to the TRIzol protocol (Invitrogen)

and then mixed using approximately the same quantity. Total mRNAs were purified from total RNA by Dynal Oilgo (dT) beads (Invitrogen). Random oligo-nucleotides and M-MuLV Reverse Transcriptase (RNase H) were used to synthesize the first cDNA strand, and then the second cDNA strand was synthesized using DNA Polymerase I and RNase H. The cDNA library with an insert size of 200-500 base pairs (bps) was targeted and purified with AMPure XP beads system (Beckman Coulter), and subsequently sequenced on an Illumina HiSeq 2000 platform. Paired-end reads were generated with a read length of 90 bps. Both cDNA library construction and Illumina sequencing were carried out by BGI-ShenZhen. The raw reads were filtered by SOAPnuke (https://github.com/BGI-flexlab/SOAPnuke) with the following criteria: 1) reads contained adaptors; 2) reads with unknown nucleotides larger than 5 %; 3) low quality reads(the rate of reads which quality value≤10 is more than 20 %). After filtering, 7.13 G clean reads obtained for gene annotation and evaluation, then clean reads were uploaded to NCBI (SRA505613).

Sequence assembly

For genome assembly, we firstly performed a 17-mer analysis with KmerFreq archived in SOAPec (version 2.02) [16] to estimate the Ma Ying azalea genome size, level of heterozygosity and repeat content of the sequenced genome by using 29 Gb clean reads from 500 and 800 bp insert size libraries. The genome size (G) of *R. delavayi* was estimated by the following formula:

$$G = N \times (L - 17 + 1)/peak\_depth$$

Where N represents the total of number of reads, L represents the length of read and peak depth refers to the most frequent reads compared to the others. The homozygous or main peak was found at a depth ~35×, the left peak and right peak indicated heterozygous site and repetitive fraction separately (Fig.2). In our calculations, N was 290,808,886, L was 100 and K_depth was 35, *R.*

delavayi genome size was estimated to be 697.94 Mb, the data used in 17-mer analysis was about 41.8× coverage of the genome (Table 2).

Fig.1 The tree sequenced in this project

Table 1 Sequencing libraries and data yields for whole genome shotgun sequencing

Table 2 Statistics of genome size estimation by 17-mer analysis. The genome size was estimated according to the formula: Genome size = N × (L-17+1)/peak_depth

Fig. 2 Kmer analysis of the *R.delavayi*genome

Then we used Platanus [17] to assemble the *Rhododendron delavayi* genome in three steps: contig-assembly, scaffolding and gap-close. In conting-assembly step, the Platanus was employed to construct de Bruijn graphs from libraries with an insert size from 170 to 800 bp, modify the graphs, and display the output sequences. In brief, Platanus increase the kmer size by the step size k_{step} (default 10) and iteratively reconstructs the graphs. A series of parameters were tested, the proper parameter (-t 20 -m 300 -u 0.2 -d 0.5 -k 41 -s 10) were chose. Assembled contigs and bubbles in the graphs were obtained from this step. In scaffolding step, the bubbles and reads from small insert size (170-800 bp) and big insert size (2 kb-40 kb) were mapped onto the assembled contigs for scaffold construction. In gap filling step, Platanus with "gap_close" parameter was took, gaps within scaffolds were filled by libraries with an insert size from 170 to 800 bp in cases where one end could be mapped to one contig and the other end extended into a gap. Two more Gap filling steps were performed based on the assembly results by utilizing KGF [18] (V1.06) and GapCloser software (v1.12-r6) [19]. To achieve optimal assembly result, Rabbit (a Poisson-based K-mer model, ftp://ftp.genomics.org.cn/pub/Plutellaxylostella/) was used to determine repeat sequences, segmental duplications or divergent haplotypes. After removing redundant sequences, a

total scaffold length of 695 Mb was generated (Table 3). The contig N50 was 61.81 Kb and the scaffold N50 was 637.82 Kb, while the length of scaffolds less than 100 bp were excluded.

The transcript assembly was carried out with Trinity (release-20130225) [20] with the following parameters: mini contig length 200 bp, min glue 3, group pairs distance 280, path reinforcement distance 85, and min kmer covage 3. The TGI Clustering Tool (TGICL) v. 2.152 [21] was used to remove redundancies and merge the Unigenes with overlap of at least 40 bp. Finally, 83,515 Unigenes were obtained with mean length 1,014 bp and N50 1727 bp.

Genome evaluation

We evaluated the completeness of the genome assembly using CEGMA [22] and BUSCO [23], which assess genome completeness using the conserved genes from the NCBI eukaryotic clusters of Orthologous Groups (KOGs) databases and Benchmarking Universal Single-Copy Orthologs, separately. CEGMA assess results indicated 95.97 % core eukaryotic genes were contained in our assembly (238 completeness of 248 core eukaryotic genes). BUSCO analysis showed 92.8 % of plants set (embryophyta_odb9, download from http://busco.ezlab.org/) were identified as complete (1337 completeness of 1440 BUSCOs). More detail information was shown in table 3. Furthermore, the Unigenes were aligned to *R.delavayi* genome using BLAT [24] with E-value=10e^{-6}, minIdentity=90 %. The alignment results indicated that the assembled genome of *R.delavayi* covered 96.98 % of the Unigenes, suggesting a high level of coverage (Table 4).

Table 3 The genome assembly of *R.delavayi*

Table 4 The gene coverage of *R.delavayi* by transcriptome data

Repeat annotation

The TRF v4.07 [25] was applied to find tandem repeat sequences with the following parameters:

Match =2, Mismatch =7, Delta = 7, PM = 80, PI = 10, Minscore = 50, MaxPerid = 2000. Total length 29,073,954 bp of the tandem repeat sequences was detected, comprising 4.18 % of the *R.delavayi* genome. Transposable elements were identified by using homology and *de novo* methods. Homology method: RepeatMasker (version 4.0.5) [26] was employed to identify transposable elements with RepBase library (version 20.04) [27], while RepeatProteinMask [26] was used to identify transposable elements against the TE protein database in RepBase. *de novo* method: (1) RepeatModeler [28] and LTR_FINDER [29] were applied to identify transposable elements in different model, respectively; (2) The result of RepeatModeler and LTR_FINDER were merged into a *de novo* repeat library; (3) RepeatMasker was employed to categorize the sequence against the *de novo* repeat library. Finally, the homology and *de novo* transposable elements of the same categories were merged by overlap. Transposable elements accounted for 51.77 % of the *R. delavayi* genome, while long terminal repeat elements (LTRs) comprised the largest proportion (37.48 %) of transposable elements (Table 5).

Table 5 Transposable elements in the *R. delavayi* genome

Gene prediction

We combined homology-based, *de novo*, and transcript alignments methods to predict protein-coding genes in the *R. delavayi* genome in the following four major steps, a detailed pipeline was shown in Figure 2.

In homology prediction step, we download gene sets of *Arabidopsis thaliana* [30], *Actinidia chinensis* [31], *Capsicum annuum* [32], *Mimulus guttatus* [33], *Solanumtuberosum* [34], and *Solanum lycopersicum* [35] from phytozome v9.1. For genes with alternative splicing variants, the longest transcript was selected to represent the gene. We aligned these homologous protein

sequences to the *R. delavayi* genome using TBLASTN [36] with E-value threshold 10^{-5}, and linked the BLAST hit result to candidate gene loci with solar using parameter “-a prot2genome2 -z” (https://sourceforge.net/p/treesoft/code/HEAD/tree/branches/lh3/solar/). Then, we extracted the candidate gene loci sequences with up and down stream 1k flanking sequence, using Genewise 2.2.0 [37] to define the intron-exon boundary. Genes with length less than 150bp or error structure (premature stop codon or frame-shifted) were excluded for further analysis.

In the *De novo* prediction step, 5,919 full-length genes were randomly selected from gene sets predicted by homology method to train the model parameters for AUGUSTUS (v3.03) [38]. Two software programs, AUGUSTUS and GENSCAN [39], were used to do *de novo* prediction on the repeat masked genome. Genes from homology and *de novo* method were combined to get non-redundant gene sets by GLEAN [40] (http://sourceforge.net/projects/glean-gene, parameters “-gff -minlen 150 -minintron 11 -maxintron 15000”). Genes with error structure or short length were filtered based on the same thresholds used for homology prediction.

In transcript alignment prediction step, the short reads from the mixed sample reported above were mapped to the *R.delavayi* genome using Tophat [41] to identify the splice junctions, and then cufflinks [42] was used to assemble transcripts with the Tophat outputs. The coding potential of these transcripts was identified using fifth-order Hidden Markov Model, which was achieved by trainGlimmerHMM [43] with gene sets used in AUGUSTUS training.

In gene sets combine step, GLEAN result and transcript assemblies were integrated to form a comprehensive gene set as following step: All-to all BLASTP analysis of protein sequences was performed between GLEAN result and transcript assemblies with an E-value cutoff $1e^{-10}$, coverage $\geq$ 90% and identity $\geq$ 90%. These transcript assemblies were add to the GLEAN results to form

UTR or alternative splice. If the transcript assemblies had no blast hit with the GLENA result, these transcript assemblies would be added to the final set as novel gene. As a result of these steps, a total of 32,938 non-redundant genes were obtained in the *R. delavayi* genome (Table 6). Meanwhile, the CEGMA and BUSCO evaluation demonstrated the annotation completeness to be 97.09 % (234 of 241 core eukaryotic genes were aligned) and 87.4 % (1,258 completeness of 1,440 BUSCOs were aligned).

Function annotation

Gene functions annotation was assigned by sequence and domains conservation. 1) Sequence conserved method: the protein sequences of *R. delavayi* were aligned to KEGG [44], SwissProt and TrEMBL (Uniprot release 201406) [45] by BLASTP archived in BLASTALL2.2.26 with E-value threshold 10^{-5}. Best-hit blast results were used to define the gene function. 2) Protein domain based method: InterProScan-5.11-51.0 [46] was employed to identify the motif and domain against the public databases Pfam [47], PRINTS [48], ProDom [49], SMART [50], PANTHER [51]. Gene Ontology identities [52] for each gene were obtained from the corresponding InterPro entry [53]. Finally, the presence of 85.91 % genes had annotation entries, with 22,946 InterPro, 6,471 GO, 21,210 KEGG, 22,693 SwissProt and 27,975 TrEMBL (Table 7).

Fig. 2 The gene model annotation pipeline.

Table 6 Summary *R.delavayi* genome annotation

Table 7 statistics for functional annotation

Conclusion

Now the order *Ericales* has two draft genome sequences of two economically important species [kiwi fruit (*Actinidia chinensis*) and *R. delavayi*]. The draft whole genome sequence of *R. delavayi*

is the first genomic resource for the family *Ericaceae*. The availability of *R. delavayi* genome sequence should streamline *de novo* assembly of other species in this genus, moreover, allow scientists to investigate interactions between environmental factors and *R. delavayi* plants at molecular level, more accurately establish the position of *R. delavayi* in the evolution tree, and facilitate locating genes or loci accounted for pharmaceutically important compounds. With more genomic resources of this genus added in databases, molecular breeding would be remarkably facilitated, which should accelerate future breeding of elite varieties.

Abbreviations

Gb: Gigabase; TE: Transposable element; GO: Gene Ontology; PE: pair end;

Acknowledgements

This project was funded by the program of Science and Technology Talents Training in Yunnan province (2016HA005), and the program of Innovative Talents Promotion by Chinese Ministry of Science and Technology (2014HE002).

Availability of supporting data

Supporting data are available in the GigaDB database [54], and the raw data were deposited in the SRA527514 with the project accession PRJNA361437 for *Rhododendron delavayi* genome.

Author’s contribution

LZ, JW and QG conceived the project. YC, LM, SL, WX, JS, LP, HY, and CZ designed sample collection and extracted the genomic DNA. PX led the genome analysis, conducted the genome assembling, and predicted gene structure and repeat sequences. All of listed authors above participated in discussion of the project and data. PX, LZ (Lu Zhang), QG, and JW co-drafted the

manuscript, and LZ (Ling Zou) helped with manuscript revision. All authors read and approved the final manuscript.

Competing interests

The authors declare that they have no competing interests.

References

1. Chamberlain D, Hyam R, Argent G, Fairweather G, Walter, KS. The genus *Rhododendron*: its classification and synonymy. Royal Botanic Garden Edinburgh. 1996.
2. Gaira KS, Rawal RS, Rawat B, Bhatt ID. Impact of climate change on the flowering of *Rhododendron arboreum* in central Himalaya, India. Current Science (00113891).2014; 106 (12).
3. Singh N. Flowering phenology of tree *Rhododendron arboretum* along an elevation gradient in different sites of Kumaun Himalayas. International journal of science and nature. 2014; 5: 572-576.
4. Bi Y, Xu J, Yang J, Li Z, Gebrekirstos A, Liang E, Yang X. Ring-widths of the above tree-line shrub *Rhododendron* reveal the change of minimum winter temperature over the past 211 years in Southwestern China. Climate Dynamics. 2016; 1-15.
5. Komac B, Esteban P, Trapero L, Caritg R. Modelization of the Current and Future Habitat Suitability of *Rhododendron ferrugineum* Using Potential Snow Accumulation. PloS one. 2016; 11(1): e0147324.
6. Zha HG., Milne RI, Sun H. Morphological and molecular evidence of natural hybridization between two distantly related *Rhododendron* species from the sino-himalaya. Botanical Journal of the Linnean Society, 2008; 156(1): 119-129.
7. Yu SX. Research on the problem on the problems of classification of the genus *Rhododendron*. Journal of Wuhan Botanical Research. 1986; 24(3): 161-164.
8. Zha HG, Milne RI, Sun H. Asymmetric hybridization in *Rhododendron agastum*: a hybrid taxon comprising mainly F1s in Yunnan, China. Annals of Botany. 2009; mcp267.
9. Zhang JL, Zhang CQ, Gao LM, Yang JB, Li HT. Natural hybridization origin of *Rhododendron agastum* (Ericaceae) in Yunnan, China: inferred from morphological and molecular evidence. Journal of plant research. 2007; 120(3): 457-463.
10. Ming TL, Fang RC, The phylogeny and evolution of genus *Rhododendron*, Acta Botanica Yunnanica, 1990; 12(4): 353-365.
11. Zhou W, Oh J, Li W, Kim DW, Yang MH, Jang JH, Ahn SJ, Lee HS, Ma, M. Chemical constituents of the Korean endangered species *Rhododendron brachycarpum*. Biochemical Systematics and Ecology. 2014; 56: 231-236.
12. Qiang Y, Zhou B, Gao K. Chemical constituents of plants from the genus *Rhododendron*. Chemistry & Biodiversity. 2011; 8(5): 792-815.
13. Zha HG, Milne RI, Sun H. Morphological and molecular evidence of natural hybridization between two distantly related *Rhododendron* species from the Sino-Himalaya. Botanical Journal of the Linnean Society. 2008; 156(1): 119-129.
14. Fang MY, Fang RZ, He MY, Hu LZ, Yang HB, Chamberlain DF. *Rhododendron*. In: Wu ZY, Raven PH. eds. Flora of China, vol. 14. Beijing and St Louis, Science Press and Missouri Botanical Garden. 2005; 260–455.
15. Sharma A, Poudel RC, Li A, Xu J, Guan K. Genetic diversity of *Rhododendron delavayi* var. *delavayi* (CB Clarke) Ridley inferred from nuclear and chloroplast DNA: implications for the conservation of fragmented populations. Plant systematics and evolution. 2014; 300(8): 1853-1866.
16. Murray MG, Thompson WF. Rapid isolation of high molecular weight plant DNA. Nucleic Acids Research. 1980; 8: 4321-4325.
17. Li RQ, Wei F, Tian G, Zhu HM, Lin H, Cai J, Huang QF, Cai QL, Li B, Bai Y, et al. The sequence and *de novo* assembly of the giant panda genome. Nature. 2010; 463: 311-317.
18. Kajitani R, Toshimoto K, Noguchi H, Toyoda A, Ogura Y, Okuno M, Yabana M, Harada M, Nagayasu E, Maruyama H, et al. Efficient de novo assembly of highly heterozygous genomes from whole-genome shotgun short reads. Genome Research. 2014; 24(8): 1384-95.
19. Short Oligonucleotide Analysis Package homepage: http://soap.genomics.org.cn/. Accessed 16 June 2016.
20. Luo RB, Liu BH, Xie YL, Li ZY, Huang WH, Yuan JY, He GZ, Chen YX, Pan Q, Liu YJ, et al. SOAPdenovo2: an empirically improved memory-efficient short-read de novo assembler. GigaScience. 2012; 1: 12.

21. Grabherr MG, Haas BJ, Yassour M, Levin JZ, Thompson DA, Amit I, Adiconis A, Fan L, Raychowdhury R, Zeng Q, et al. Full-length transcriptome assembly from RNA-Seq data without a reference genome. Nat Biotechnology. 2011; 29(7): 644-52.

22. Pertea G, Huang XQ, Liang F, Antonescu V, Sultana R, Karamycheva S, Lee Y, White J, Cheung F, Parvizi B, et al. TIGR gene indices clustering tools (TGICL): a software system for fast clustering of large EST datasets. Bioinformatics. 2003; 19(5): 651-652.

23. Parra G, Bradnam K, Korf I. CEGMA: a pipeline to accurately annotate coregenes in eukaryotic genomes. Bioinformatics. 2007; 23: 1061-7.

24. Simão FA, Waterhouse RM, Ioannidis P, Kriventseva EV, Zdobnov EM. BUSCO: assessing genome assembly andannotation completeness with single copy orthologs. Bioinformatics. 2015; 31(19): 3210-3212.

25. Kent WJ. BLAT the BLAST like alignment tool. Genome Research. 2002; 12: 656-664.

26. Benson G, Tandem repeats finder: a program to analyze DNA sequence. Nucleic Acid Res. 1999; 27: 573-580.

27. Chen NS. Using RepeatMasker to identify repetitive elements in genomic sequences. Curr Protoc Bioinformatics. 2009; Chapter 4: Unit 4.10.

28. Jurka J, Kapitonov VV, Pavlicek A, Klonowski P, Kohany O, Walichiewicz J. Repbase Update, a database of eukaryotic repetitive elements. Cytogenet Genome Research. 2005; 110: 462-467.

29. Abrusán G, Grundmann N, DeMester L, Makalowski W. TEclass—a tool forautomated classification of unknown eukaryotic transposable elements. Bioinformatics. 2009; 25: 1329–30.

30. Xu Z, Wang H. LTR_FINDER: an efficient tool for the prediction of full-length LTR retrotransposons. Nucleic Acids Res. 2007; 35: W265-268.

31. Kaul S, Koo HL, Jenkins J, Rizzo M, Rooney T, Tallon LJ, Feldblyum T, Nlerman W, Benlto MI, Lin XY, et al. Analysis of the genome sequence of the flowering plant *Arabidopsis thaliana*. Nature. 2000; 408: 796-815.

32. Huang S, Ding J, Deng D, Tang W, Sun H, Liu D, Yu J. Draft genome of the *kiwifruit Actinidiachinensis*. Nature Communications. 2013; 4: 2640. doi: 10.1038/ncomms3640.

33. Qin C, Yu C, Shen Y, Fang X, Chen L, Min J, Yang Y. Whole-genome sequencing of cultivated and wild peppers provides insights into Capsicum domestication and specialization. Proceedings of the National Academy of Sciences. *2*014; 111(14): 5135-5140.

34. https://phytozome.jgi.doe.gov/pz/portal.html#!info?alias=Org_Mguttatus

35. The Potato Genome Sequencing Consortium: Genome sequence and analysis of the tuber crop potato. Nature. 2011; 475: 189-195.

36. Altschul SF, Madden TL, Schaffer AA, Zhang JH, Zhang Z, Miller W, Lipman DJ. Gapped BLAST and PSI-BLAST: a new generation of protein database serch programs. Nucleic Acids Research. 1997; 25: 3389-3402.

37. Birney E, Clamp M, Durbin R. GeneWise and Genomewise. Genome Research. 2004; 14: 988-995.

38. Stanke M, Keller O, Gunduz I, Hayes A, Waack S, Morgenstern B. AUGUSTUS: ab initio prediction of alternative transcripts. Nucleic Acid Res. 2006; 34: W435-439.

39. Burge C, Karlin S. Prediction of complete gene structures in human genomic DNA. Journal of Molecular Biology. 1997; 268: 78-94.

40. Elsik CG, Mackey AJ, Reese JT, Milshina NV, Roos DS, Weinstock GM. Creating a honey bee consensus gene set. Genome Biology. 2007; 8: R13.

41. Trapnell C, Pachter L, Salzberg SL. TopHat: discovering splice junctions with RNA-Seq. Bioinformatics. 2009; 25: 1105-1111.

42. Trapnell C, Williams BA, Pertea G, Mortazavi A, Kwan G, van Baren MJ, Salzberg SL, Wold BJ, Pachter L.Transcript assembly and quantification by RNA-Seq reveals unannotated transcriptsand isoform switching during cell differentiation. Nature Biotechnology. 2010; 28: 511–5.

43. Majoros WH, Pertea M, Salzberg SL. TigrScan and GlimmerHMM: two open source ab initio eukaryotic gene-finders. Bioinformatics. 2004; 20(16): 2878–9.

44. Ogata H, Goto S, Sato K, Fujibuchi W, Bono H, Kanehisa M. KEGG: Kyoto Encyclopedia of Genes and Genomes. Nucleic Acids Res. 1999; 27: 29-34.

45. Bairoch A, Apweiler R. The SWISS-PROT protein sequence database and its supplement TrEMBL in 2000. Nucleic Acid Res. 2000; 28: 45-48.

46. Zdobnov EM, Apweiler R. InterProScan-an integration platform for the signature-recognition methods in InterPro. Bioinformatics. 2001; 17: 847-848.

47. Bateman A, Birney E, Durbin R, Eddy SR, Howe KL, Sconnhammer EL. The Pfam protein families database. Nucleic Acids Research. 2000; 28: 263-266.

48. Attwood TK, Cronig MD, Flower DR, Lewis AP, Madey JE, Scordis P, Selley JN, Wright W. PRINTS-S: the database formerly known as PRINTS. Nucleic Acids Res. 2000; 28: 225-227.

49. Corpet F, Gouzy J, Kahn D. Recent improvements of the ProDom database of protein domain families. Nucleic Acids Res. 1999: 27: 263-267.

50. Schult J, Copley RR, Doerks T, Ponting CP, Bork P. SMART: a web-based tool for the study of genetically mobile domains. Nucleic Acids Res. 2000; 28: 231-234.

51. Mi HY, Lazareva-Ulitsky B, Loo R, Kejariwal A, Vandergriff J, Rabkin S, Guo N, Muruganujan A, Doremieux O, Campbell MJ, et al. The PANTHER database of protein families, subfamilies, functions and pathways. Nucleic Acids Research. 2005; 33: 284-288.

52. Ashburner M, Ball CA, Blake JA, Botstein D, Butler H, Cherry JM, Davis AP, Dolinski K, Dwight SS, Eppig JT. Gene Ontology: tool for the unification of biology. Nature Genetics. 2000; 25(1): 25–9.

53. Burge S, Kelly E, Lonsdale D, Mutowo-Muellenet P, McAnulla C, Mitchell A. Manual GO annotation of predictive protein signatures: the InterPro approach to GO curation. Database. 2012; 2012: 257-264.

54.

 Click here to download Table summplement table.docx

Table 1 Sequencing libraries and data yields for whole genome shotgun sequencing

Library type	Lane	Reads	Insert Size	Raw bases		Clean bases	
				Total bases	Depth(X)	Total bases	Depth(X)
PE101	2	100	170	80.47		74.12	
PE151	1	150	250	59.69		47.20	
PE101	4	100	500	47.89		43.58	
PE101	3	100	800	42.22		36.79	
PE50	2	49	2000	30.36		19.56	
PE50	3	49	5000	23.11		9.06	
PE50	3	49	10000	20.17		6.71	
PE50	2	49	20000	19.01		4.35	
PE50	1	49	40000	13.91		4.69	

Note: Sequencing depth was calculated based on a genome size of 697Mb. High-quality data were obtained by filtering raw data for low-quality and duplicate reads.

Table 2 Statistics of genome size estimation by 17-mer analysis. The genome size was estimated according to the formula: Genome size = N×(L-17+1)/peak_depth

Genome	Kmer length(bp)	Kmer number	Kmer depth	Estimated Genome size	Read numbers	Genome coverage
Rhododendron delavayi	17	24,427,946,424	35	697,941,326	290,808,886	41.8×

Table 3 The genome assembly and completeness of *R. delavayi*

Assembly	Length(bp)	Continuity			CEGMA		BUSO	
		N10	N50	N90	C	P	C	F
Contigs	657,780,215 (209,969)	191,762 (252)	61,801 (2871)	7,447 (13,555)				
Scaffolds	695,092,854 (193,091)	1,819,505 (31)	637,826 (313)	71,928 (1,363)	95.97% [238]	98.39% [244]	92.8% [1337]	1.8% [26]

Note: Numbers of contigs/scaffolds are shown in parentheses. Numbers of genes that match CEGMA or BUSO are shown in square brackets. CEGMA: C means completeness, P means partial; BUSO: C means completeness, F means fragment;

Table 4 The gene coverage of *R. delavayi* by transcriptome data

Dataset	Number	Total length (bp)	Base coverage by assembly (%)	Sequences coverage by assembly (%)
All	83,515	84,701,674	96.98	99.53
>200bp	83,515	84,701,674	96.98	99.53
>500bp	46,582	73,471,401	96.90	99.79
>1000bp	29,816	61,377,043	96.80	99.88

Table 5 Transposable elements in the *R. delavayi* genome

	Repabse TEs	Protein TEs	*De novo* TEs	Combined TEs	
	length	length	length	length	percentage
DNA	7,882,501	7,328,645	69,812,249	77,776,557	11.19
LINE	4,811,976	12,454,813	31,065,638	36,834,088	5.30
SINE	125,792	0.00	869,547	991,785	0.14
LTR	34,884,681	52,469,776	257,040,066	260,532,496	37.48
Other	552	0.00	0.00	552	0.00
Unknown	0.00	0.00	4,565,754	4,565,754	0.67
Total	470,018,44	72,016,848	350,372,642	359,874,503	51.77

Note: Repabse TEs means RepeatMask against Repbase; Protein TEs means RepeatProteinMask result against Repbase protein; *De novo* TEs means RepeatMask against the *de novo* library; Combined TEs means the combine result of three steps.

Table 6 Summary *R. delavayi* genome annotation

Gene set		Numbers	Average gene length (bp)	Average CDS length (bp)	Average exon per gene	Average exon length (bp)	Average intron length (bp)
De novo	AUGUSTUS	42,67	2623.41	974.42	4.76	204.56	438.16
	GENSCAN	35,85	11242.68	1186.91	6.35	186.87	1879.03
Homolog	*A.chinensis*	45,44	3501.48	846.20	3.21	263.43	1200.29
	A.thaliana	31,95	3724.50	994.90	4.07	244.30	888.41
	C.annuum	47,67	2558.30	805.26	3.01	267.50	872.00
	M.guttatus	34,61	3454.51	963.76	3.95	244.21	845.35
	S.lycopersicu	38,80	3324.95	917.11	3.74	245.47	880.01
	S.tuberosum	39,08	2958.21	850.18	3.22	263.79	948.30
	GLEAN	29,58	4126.65	1150.32	4.84	237.78	775.53
	Transcriptome	38,27	2989.97	828.78	3.45	240.07	881.29
	Final set	32,93	4434.22	1153.21	4.62	249.70	785.08

Table 7 statistics for functional annotation

	Numbers	Percent (%)
InterPro	22,946	69.66
GO	16,471	50.00
KEGG	21,210	64.39
Swissprot	22,693	68.90
TrEMBL	27,975	84.93
Annotated	28,296	85.91
Unanotated	4,642	14.09

Figure1 Click here to download Figure Fig1 Rhododendron tree.pdf

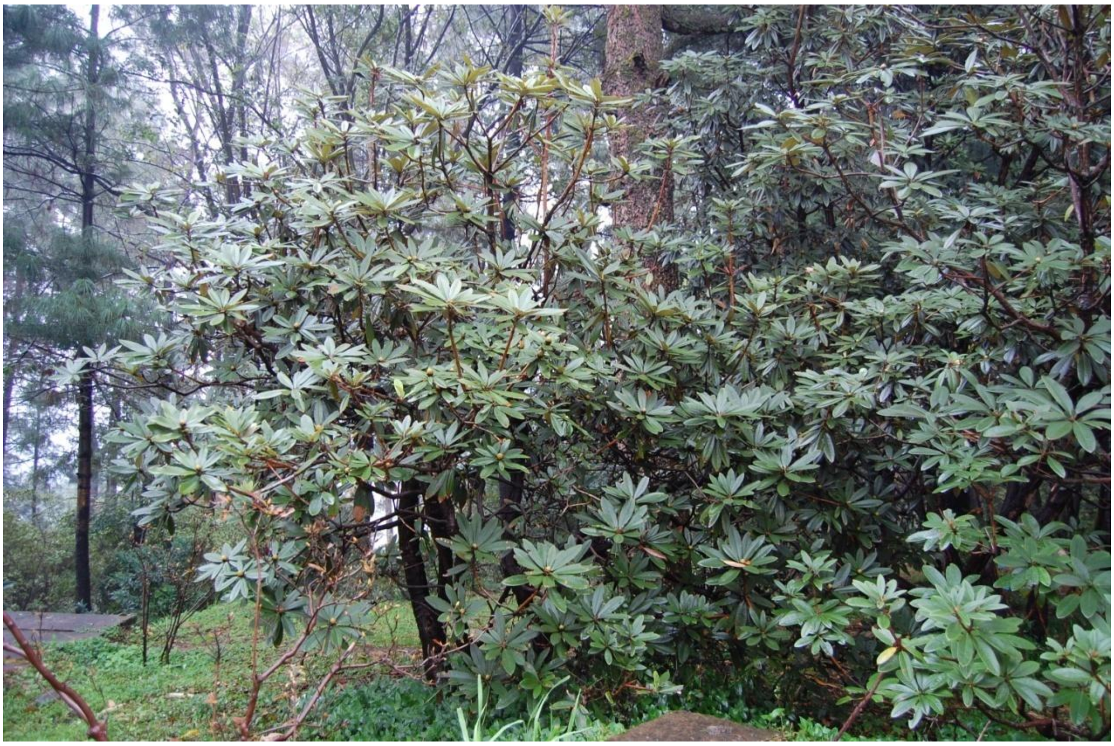

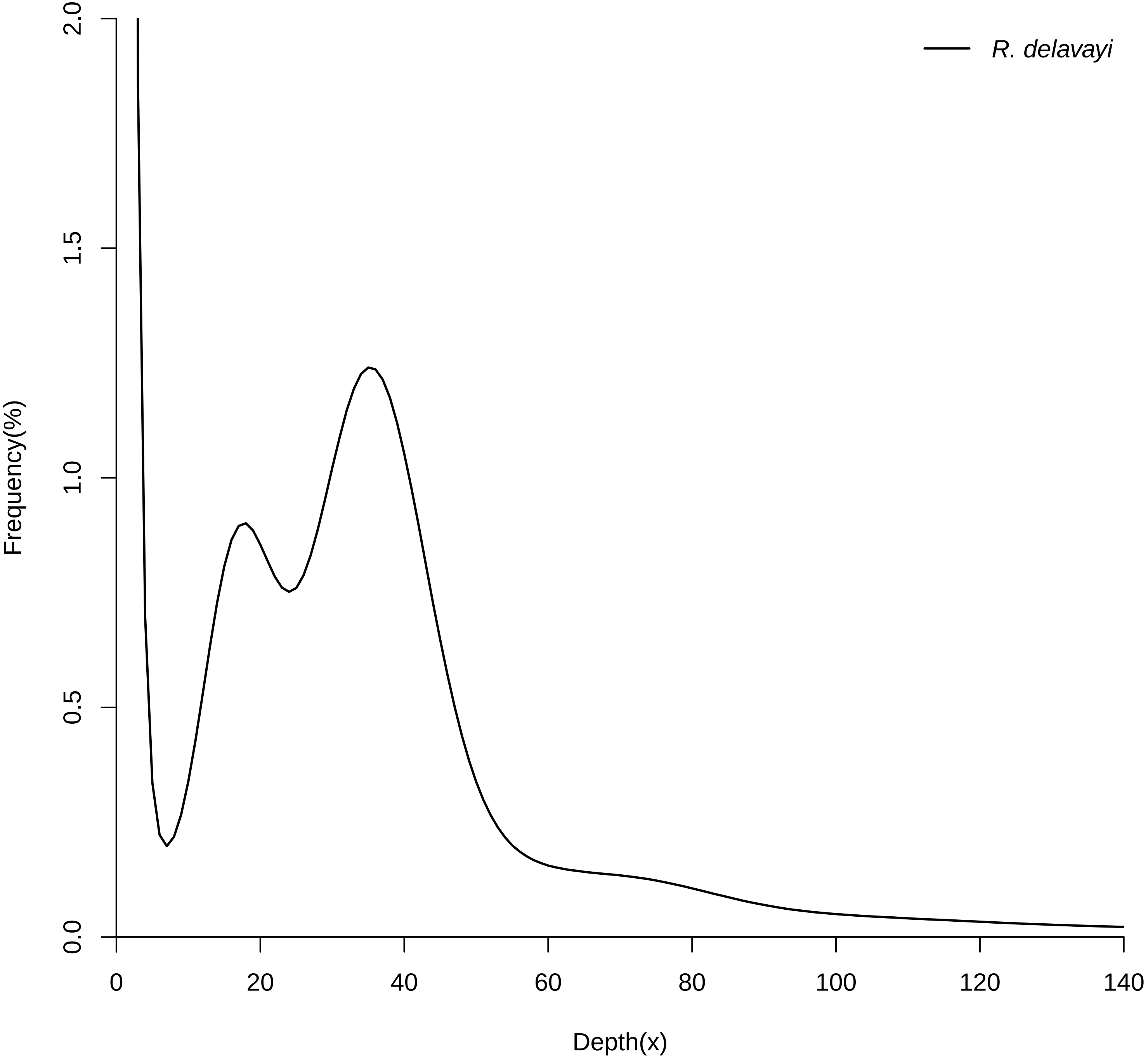
R. delavayi
Frequency(%)
2.0
1.5
1.0
0.5
0.0
0
20
40
60
80
100
120
140
Depth(x)

 Click here to download Figure Fig.3.pdf

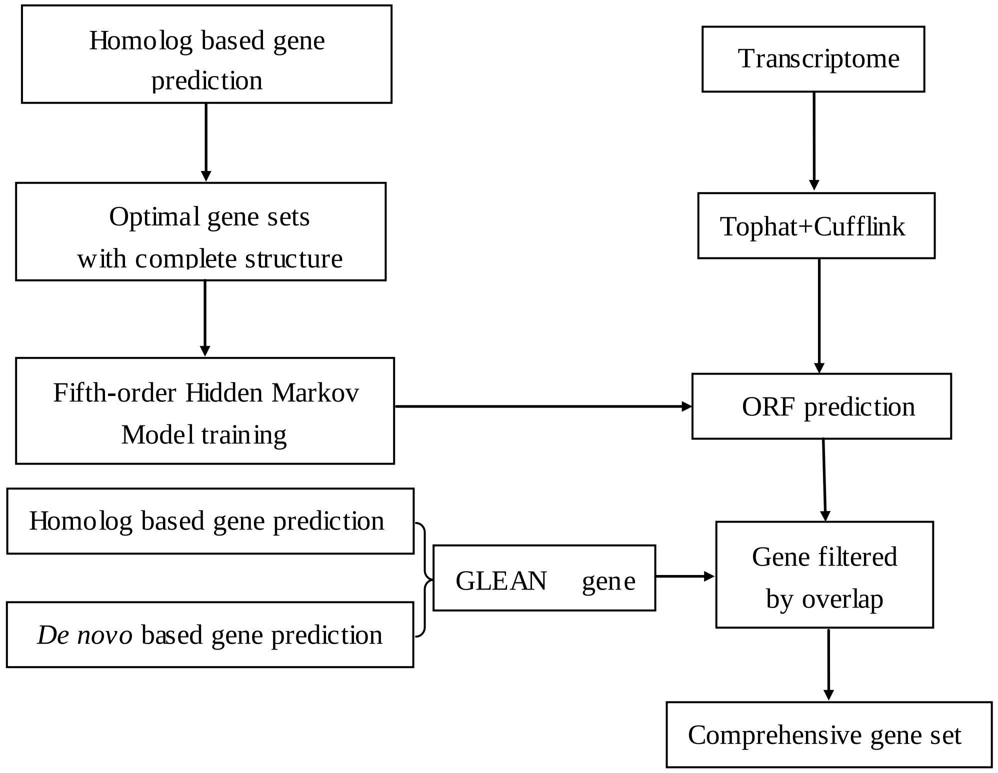

Supplement: GIGA-D-17-00027_Original-Submission.pdf [file gix076_GIGA-D-17-00027_Original-Submission.pdf]
