# Supplementary material for: The draft genome assembly of Rhododendron delavayi Franch. var. delavayi
Source: Gigascience. 2017 Aug 26;6(10):1–11. doi: 10.1093/gigascience/gix076 (PMC5632301; doi:10.1093/gigascience/gix076)

Manuscript

Click here to download Manuscript 20170529 Revised text_TM-Z.docx

The draft genome assembly of *Rhododendron delavayi* Franch. var. *delavayi*

Lu Zhang[1,2†], Pengwei Xu[3†], Yanfei Cai[1,2†], Lulin Ma[1,2†], Shifeng Li[1,2], Shufa Li[1,2], Weijia Xie[1,2], Jie Song[1,2], Lvchun Peng[1,2], Huijun Yan[1,2], Ling Zou[1,2], Yongpeng Ma[4], Chengjun Zhang[5], Qiang Gao[3*], Jihua Wang[1,2*]

Abstract

Rhododendron delavayi Franch. is globally famous as an ornamental. Its distribution in southwest China covers several different habitats and environments. However, not much research had been conducted on *Rhododendron* spp. at molecular level, which hinders understanding of the nature of its wide adaptability to different environments, evolution, speciation and synthesis of secondary metabolites. Here, we report genome assembly and gene annotation of *R. delavayi* var. *delavayi* (the second genome sequenced in the Ericaceae), which will facilitate the study of the family, and have further applications in genome-assisted cultivar breeding.

Findings: The final size of the assembled *R. delavayi* var. *delavayi* genome (695.09 Mb) was close to the 697.94 Mb estimated by k-mer analysis. A total of 336.83 gigabases (Gb) of raw Illumina HiSeq 2000 reads were generated from nine libraries (with insert sizes ranging from

[*]Correspondence: wjh0505@gmail.com; gaoqiang@genomics.cn

[†] Equal contributors

[1] Flower Research Institute of Yunnan Academy of Agricultural Sciences, National Engineering Research Center For Ornamental Horticulture, Kunming 650205, China

[2] Key Lab of Yunnan Flower Breeding, Kunming 650205, China

[3] BGI-Shenzhen, Shenzhen 518083, China

[4] Kunming Botanical Garden, Kunming Institute of Botany, Chinese Academy of Science, Kunming 650204, China

[5] Germplasm Bank of Wild species, Kunming Institute of Botany, Chinese Academy of Science, Kunming 650204, China

170bp to 40kb), achieving a raw sequencing depth of 482.61×. After quality filtering, 246.06 Gb of clean reads were obtained, giving 352.55× coverage depth. Assembly using Platanus gave a total scaffold length of 695.09 Mb, with a contig N50 of 61.8 kb and a scaffold N50 of 637.83kb. Gene prediction resulted in the annotation of 32,938 protein-coding genes. The genome completeness was evaluated by CEGMA and BUSCO, and reached 95.97 % and 92.8 % respectively. The Gene annotation completeness was also evaluated by CEGMA and BUSCO, and reached 97.01 % and 87.4 %. Genome annotation revealed that 51.77 % of the *R. delavayi* genome is composed of transposable elements, and 37.48 % of long terminal repeat elements (LTRs).

Conclusions: The *de novo* assembled genome of *R. delavayi* var. *delavayi* (hereinafter referred to as *R. delavayi*) is the second genomic resource of the family *Ericaceae*, and will provide a valuable resource for research on future comparative genomic studies in *Rhododendron* species. The availability of the *R. delavayi* genome sequence will hopefully provide a tool for scientists to tackle open questions regarding molecular mechanisms underlying environmental interactions in the genus *Rhododendron*, more accurately understand the evolutionary processes and systematics of the genus, facilitate the identification of genes encoding pharmaceutically important compounds, and accelerate molecular breeding to release elite varieties.

Keywords: *Rhododendron delavayi*, Genomics, Genome assembly, Annotation

Background

Rhododendron L. is a genus in the family Ericaceae. It is one of the largest and most diverse genera in the family and is distributed predominantly throughout the Northern hemisphere, but also reaching into the asian tropics. Over 1000 species of *Rhododendron* are currently recognized, of which 567 species representing 6 subgenera are known from China. Of these Chinese species

approximately 80% are endemic [1, 2]. Because of the adaptability of this genus to different environments, species such as *R. arboreum* and *R. ferrugineum* have been used to investigate the effects of different environmental factors on plant growth, development, and domestication [3, 4, 5, 6].

Certain secondary metabolites in *Rhododendron*, have been investigated in connection with antioxidant, anti-inflammatory, anti-carcinogen, and anti-bacterial properties; these compounds have potential in the alleviation of symptoms in conditions including diabetes, arthritis, headache and hypertension [7, 8, 9]. Genome-level sequencing could help investigation into genes responsible for these metabolites, and could facilitate the characterization of bio-active compounds and down-stream production.

Most species of *Rhododendron* are diploid (2n = 26). The relatively low levels of ploidy and reported introgression of genetic material between species in nature might be important in the evolution and speciation of *Rhododendron* [10], e.g. natural interspecific hybridization is common among *R.delavayi*, *R.decorum*, *R.irroratum* and many other species. One reason why *Rhododendron* is so successful in horticulture is because the relative ease with which hybrids can be produced [11, 12]. Although previous research on morphology, anatomy and cytology of *Rhododendron* suggested that the subgenus *Hymenanthes* represents a basal state of this genus [13]. Furthermore, classification attempts employing only a small set of gene regions was not able to resolve relationships within the subgenus [14, 15].

R. delavayi Franch. is widely distributed throughout southwest China, and grows at a wide altitudinal range, between 1200 and 3200 m. The species belongs to subgenus *Hymenanthes,* subsection *Arborea* [1, 16]. Four varieties have been described for this species. *Rhododendron*

delavayi var. *peramoenum* has narrow leaves and has been reported from western Yunnan, northeast India and Myanmar, whereas *R. delavayi* var. *delavayi* has broader leaves than the former and mainly dominates in the Chinese range of the species. Another two varieties *R. delavayi* var. *adenostylum* and *R. delavayi* var. *pilostylum* were recently shown to fall within the spectrum of morphologies observed in hybrids between *R. delavayi* and *R.irroratum* [17]. In this project material obtained from *R. delavayi* var. *delavayi* was used to generate genome sequences.

Due to its very attractive flowers and good resistance to arid and cold climates, *R. delavayi* has become a highly profitable ornamental flower in the market, especially in China and some Southeastern Asian countries, such as Vietnam, Thailand, Burma and India. Nevertheless, it was believed that the anthropogenic activities have significantly reduced diversity of plants of this genus in the nature [18].

The aim of this project was to obtain a genome sequence of *R. delavayi* to facilitate future research. With an available genome sequence, several next-generation sequencing approaches requiring a reference will become feasible, which will enable more in-depth research into genome-environment interactions, help with marker development for phylogenetic studies, and open possibilities for genome-assisted cultivar breeding and other down-stream applications.

Figure1. *Rhododendron delavayi* Franch. on Cang Shan Mountain, Dali

Data description

Sample collection

Tissue samples were obtained from a 50-year old tree growing in Jindian National Forest Park (Kunming, Yunnan, Taxonomy ID: 321363). This tree was transplanted from Cang Shan Mountain (Dali, Yunnan) in 1995. For genome library preparation, only leaf tissue was used; for transcriptome sequencing, samples were obtained from five different tissues: flowers, flower buds,

young leaves, mature leaves and young stems. After collection, tissues were immediately transferred into liquid nitrogen and stored until DNA and RNA extraction.

Illumina sequencing strategy

Genomic DNA was extracted from the leaf tissue using a standard CTAB extraction [19]. Different methods were used to construct different insert size libraries. For the small-insert libraries (170, 250, 500 and 800 bp), Illumina's protocols were used as following (Illumina, San Diego, CA): 1) genomic DNA was fragmented by nebulization with compressed nitrogen gas; 2) DNA ends were polished and an adenine was added to the ends of the fragments; 3) DNA adaptors (Illumina) with a single "T" overhang at the 3' end were ligated to the DNA fragments above; 4) the ligation products were run on 2 % agarose gels, and the bands corresponding to each insert size were excised.. For the large insert libraries (2, 5, 10, 20 and 40 kb), Illumina's mate pair library protocols were followed: 1) genomic DNA was fragmented by nebulization with compressed nitrogen gas; 2) DNA ends were polished using dNTPs labeled with biotin and circularized for self-ligation; 3) circularized DNA was fragmented again by DNA Exonuclease, followed by enrichment of fragments containing biotin/streptavidin with magnetic beads; 4) fragment ends were further polished, followed by addition of an "A" base and adaptors to form the large insert libraries.

As shown in Table 1, the read length of the large insert libraries (2, 5, 10, 20 and 40 kb) was 49 bp, and the read length of the small insert libraries (170, 500 and 800 bp) was 100 bp, with the exception of the 250 bp insert library, which had a read length of 150 bp.. A total of 336.83 Gb (482.61×) raw reads were generated from all constructed libraries. Before assembly, reads with low quality, PCR duplication and adapter contaminations were filtered by SOAPfilter (as included in SOAPdenovo, v2.04) [20], and finally 246.06 Gb (352.55×) high-quality sequences were

obtained for genome assembly.

Table 1. Sequencing libraries and data yields for whole genome shotgun sequencing

RNA of each tissue was extracted separately according to the TRIzol protocol (Invitrogen) and then combined them in homogenized RNA concentration. Total mRNAs were purified from total RNA by Dynal Oilgo (dT) beads (Invitrogen). Random oligo-nucleotides and M-MuLV Reverse Transcriptase (RNase H) were used to synthesize the first cDNA strand, and then the second cDNA strand was synthesized using DNA Polymerase I and RNase H. The cDNA libraries with insert sizes of 200-500 base pairs (bps) were selected and purified with the AMPure XP beads system (Beckman Coulter), and subsequently sequenced on an Illumina HiSeq 2000 platform. Both cDNA library construction and Illumina sequencing were carried out by BGI-ShenZhen. Paired-end reads were generated with a read length of 90 bps. The raw reads were filtered by SOAPnuke (https://github.com/BGI-flexlab/SOAPnuke) with the following criteria for been discarded: 1) reads contained adaptors; 2) reads with unknown nucleotides larger than 5 %; 3) low quality reads (the rate of reads which quality value$\leqslant$10 is more than 20 %). After filtering, 7.13 G clean reads were obtained for genome evaluation and gene annotation. All clean reads were uploaded to NCBI (SRA505613).

Genome size estimates

We characterized the genome sequence (genome size, heterozygosity and repetitive content) using the distribution of k-mers of length 17, 21, 25 and 27 from the clean reads (29 Gb clean reads from 500 and 800 bp insert size libraries). This analysis was performed using KmerFreq (included in SOAPdenovo, v2.04). The genome size (G) of *R. delavayi* was estimated by the following formula: G = *k*-mer_number/*k*-mer_depth, where the *k*-mer_number is the total number

of *k*-mers, and *k*-mer_depth refers to the most frequent peak.

All four *k*-mer distribution curves displayed four distinct peaks (Figure 2A). The first peak at *k* = 1 was an artifact caused by sequencing errors, each of which created a *k*-mer that never occured in the genome. The remaining three peak distributions indicated that the genome is a slightly repetitive, heterozygous, diploid genome. The third peak was a "diploid" peak (*k*-mers shared between homologous chromosomes), and was twice as deep as the second "haploid" peak (*k*-mers unique to a haplotype due to heterozygosity). The fourth peak was a repetitive peak (k-mers duplicated due to repetition), and was twice as deep as the "diploid" peak. For *k* = 17, the homozygous peak (the third peak) was found at a depth of ~35×, with a k-mer_number of 24,427,946,424 and k-mer_depth of 35. The *R. delavayi* genome size was estimated to be 695.94 Mb, and the data used in 17-mer analysis was about 41.7× coverage of the genome. All the k-mer sizes yielded similar genome size estimates of ~697-717 Mb (Table 2).

Table 2. Statistics of genome size estimation by KmerFreq with k = 17, 21, 25 and 27

We also used jellyfish v2.0 [21] to make *k*-mer histograms for k-mers 25 and 31 (Figure 2B), and genome size estimates were 693 and 703Mb, respectively (Table 3). The *k*-mer distribution obtained by jellyfish showed a similar trend to KmerFreq. Using the result from jellyfish as input for GenomeScope (http://qb.cshl.edu/genomescope/), heterozygosity estimates for the *R. delavayi* genome were in the range of ~0.9-1.1%.

Table 3. Properties of the *R. delavayi* k-mer distributions for k = 25 and k =31 using jellyfish

Figure 2. *k*-mer analysis of the *R. delavayi* genome. (A) Histograms of *k*-mer frequencies in the clean read data for *k* = 17 (green), *k* = 21 (purple), *k* =25 (orange) and *k* = 27 (yellow) by KmerFreq. (B) Histograms of *k*-mer frequencies in clean data for *k* = 25 (red) and *k* = 31 (blue) by jellyfish. The x -axis shows the number of times a *k*-mer occurred; *e.g.* the peaks near x = 31 indicate the number of *k*-mers that occurred 31 times in the data.

Genome and transcriptome assembly

The *Rhododendron delavayi* genome was assembled using platanus v1.2.4 [20], employing the three following steps: contig-assembly, scaffolding and gap-closing. For the contig-assembly step, the command line parameters 'platanus assemble -t 20 -m 300 -u 0.2 -d 0.5 -k 41 -s 10' were specified to construct de Bruijn graphs for small insert size libraries (170, 250, 500, and 800 bp), to modify the graphs, and display the output sequences. With these options, Platanus increased the *k*-mer size by the step size k_{step} (default 10) and iteratively reconstructs the graphs. Assembled contigs and bubbles in the graphs were obtained from this step. In the scaffolding step, the bubbles and reads from the libraries with small insert sizes (170, 250, 500, and 800 bp) and large insert sizes (2, 5, 10 20, and 40 kb) were mapped onto the assembled contigs for scaffold construction. The command used for this was "platanus scaffold -t 20 -u 0.2 -c contigs.fasta -b bubble.fasta -IP 'reads from small insert size libraries' -OP 'reads from large insert size libraries' ". In the gap filling step, the command used was "platanus gap_close –t 20 –IP 'reads from small insert size libraries' ", and gaps within scaffolds were filled by reads from small insert size libraries where one end could be mapped to one contig and the other end extended into a gap. Two more gap filling steps were performed based on the assembly results, first utilizing KGF [22] (v1.06), followed by GapCloser (v1.12-r6) [22].

To remove probable redundant sequence in the genome, we used jellyfish v2.0 to calculate the 17-mer frequency table from all short insert libraries, then passed the result to trimDup, which comes as part of Rabbit [23] (ftp://ftp.genomics.org.cn/pub/Plutellaxylostella/Rabbit_linux-2.6.18-194.blc.tar.gz). The following command was used 'trimDup 17-mer_table 17 1.5*main_peak genome.fa 0.3'. Hence, *k*-mers were excluded, if their frequency was higher than 1.5 times the main peak. Each *k*-mer was defined as either a 'repeat' or a 'unique' *k*-mer, depending on whether

its occurrence frequency was greater or less than twice the average frequency. Rabbit uses a Poisson-based *k*-mer model to establish a 17-mer frequency table from each scaffold of the genome sequences, and then determines unique *k*-mers belonging to each scaffold and common *k*-mers shared by the scaffolds. The 17-mer frequency table generated in jellyfish is then used to filter the scaffolds so that the ratio of common to unique *k*-mers reaches 0.3. After the removal of 57.52 Mb redundant scaffolds, a total scaffold length of 695 Mb was generated (Table 4). The contig N50 was 61.81 Kb and the scaffold N50 was 637.82 Kb, while the scoffolds with lengths less than 100 bp were excluded. Meanwhile, we also ran another *de novo* assembler, SOAPdenovo2, with various modifications of parameters, but the results (Table 5) from SOAPdenovo2 were not better than those generated above.

Table 4. The genome assembly and completeness of *R. delavay*

Table 5. Statistics of the assembly with different parameters.

Transcript assembly was carried out in Trinity (release-20130225) [24] with the following parameters: minimum contig length 200 bp, min glue 3, group pairs distance 280, path reinforcement distance 85, and min kmer covage 3. The TGI Clustering Tool (TGICL) v.2.1 [25] was used to remove redundancies and merge the Unigenes with overlaps of at least 40 bp. Finally, a total of 83,515 Unigenes were obtained, with a mean length of 1,014 bp and an N50 of 1,727 bp.

Genome evaluation

We evaluated the completeness of the genome assembly using CEGMA (v2.5) [26] and BUSCO (v2.0) [27], which assess genome completeness using the conserved genes from the NCBI eukaryotic clusters of Orthologous Groups (KOGs) databases, and Benchmarking Universal Single-Copy Orthologs, respectively. CEGMA results indicated that 95.97 % of core eukaryotic genes were contained in our assembly (238 out of 248 core eukaryotic genes). BUSCO analysis

resulted in 92.8 % of plants set (embryophyta_odb9, download from http://busco.ezlab.org/) were identified as complete (1337 out of 1440 BUSCOs). More detailed information is given in table 4. The Unigenes were aligned to the *R. delavayi* genome using BLAT (v0.36) [28] with default parameters. The alignment indicated that the assembled genome of *R. delavayi* covered 96.98 % of the Unigenes, 89.57% of the Unigenes with at least 90 % coverage in one scaffold, and 98.90% of the Unigenes with at least 50 % coverage in one scaffold, suggesting a high level of coverage (Table 6).

Table 6. The gene coverage of *R. delavayi* by transcriptome data

Repeat annotation

To identify tandem repeats TRF v4.07 [29] was used with the following parameters: Match =2, Mismatch =7, Delta = 7, PM = 80, PI = 10, Minscore = 50, MaxPerid = 2000. In total 29,073,954 bp of tandem repeat sequences were detected, representing 4.18 % of the *R. delavayi* genome. Transposable elements were identified by using homology and *de novo* methods. Homology : RepeatMasker (v4.0.5) [30] was employed to identify transposable elements with RepBase library (version 20.04) [31], while RepeatProteinMask (v4.05) [30] was used to identify transposable elements against the TE protein database in RepBase. *De novo*: (1) RepeatModeler (v1.07) [32] and LTR_FINDER (v1.05) [33] were used to identify transposable elements; (2) The results from RepeatModeler and LTR_FINDER were merged into a *de novo* repeat library; (3) RepeatMasker was employed to categorize the genome sequence against the *de novo* repeat library. Finally, transposable elements identified by homology or *de novo* library within the same category were merged by overlap. Transposable elements accounted for 51.77 % of the *R. delavayi* genome, while long terminal repeat elements (LTRs) represented the largest fraction (37.48 %) of

transposable elements (Table 7). The most abundant subtypes were *Copia* and *Gypsy*, representing 6.84 % and 25.49 % of the assembly genome respectively.

Table 7. Transposable elements in the *R. delavayi* genome

Gene prediction

We combined homology-based, *de novo*, and transcript alignment methods to predict protein-coding genes in the *R. delavayi* genome. Four major steps were employed, and a detailed pipeline is given in Figure 3.

Figure 3. The gene prediction pipeline

For gene prediction based on homology, we obtained gene sets from *Arabidopsis thaliana* [34], *Actinidia chinensis* [35], *Capsicum annuum* [36], *Mimulus guttatus* [37], *Solanum tuberosum* [38], and *Solanum lycopersicum* [39]. For genes with alternative splicing variants, the longest transcript was selected to represent the gene. We aligned these homologous protein sequences to the *R. delavayi* genome using TBLASTN (v 2.2.26) [40] employing an E-value threshold of 1e-5. The resulting BLAST hits were linked to candidate gene loci using solar (v0.9.6) [41] with options "-a prot2genome2 -z". Then, we extracted the candidate gene locus sequences including 1 kb of flanking DNA upstream and downstream, used Genewise (v2.2.0) [42] to define the intron-exon boundary. Genes with lengths under 150 bp or with erroneous structure (premature stop codon or frame shifts) were excluded from further analysis.

For the *De novo* prediction step the repeat masked genome was used as input for two programs, AUGUSTUS (v3.03) [43] and GENSCAN (v1.0) [44]. To obtain a training-set for AUGUSTUS, we randomly selected 5,919 full-length genes that had been predicted based on homology, while for GENSCAN Arabidopsis parameters were used. For the final non-redundant

gene set, genes predicted based on both homology and *de novo* methods were combined with GLEAN (v1.0) [45], setting options "-gff -minlen 150 -minintron 11 -maxintron 15000". Genes with erroneous structure or of short length were again excluded based on the same thresholds used for homology prediction.

For the transcript alignment prediction step, the short reads from the transcriptome dataset generated in the previous step were mapped to the *R. delavayi* genome using Tophat (v2.1.1) [46] to identify the splice junctions. Cufflinks (v2.2.1) [47] was then used to assemble transcripts from the Tophat outputs. The coding potential of these transcripts was identified by using the same gene sets with a fifth-order Hidden Markov Model, which was achieved by the same gene sets used in the training of AUGUSTUS.

In the gene set combination step, outputs from GLEAN were combined with transcript assemblies as follows: Firstly, translated sequences of both sets were cross-matched with an all-to-all BLASTP using an E-value cutoff of 1e-10. The matching transcript assemblies were then added to the GLEAN results as either (untranslated region) UTRs or alternative splice forms, based on whether coverage and identity of the alignment results was larger than 0.9 or not. The transcript assemblies that had no BLAST hit with the GLEAN results were added to the final set as novel genes.

As a result of these steps, a total of 32,938 non-redundant genes were predicted in the *R. delavayi* genome (Table 8). These genes were scattered over 2,149 scaffolds, averaging 15.33 genes per scaffold.

We also used Maker-P [48] to predict gene model with current homolog, *de novo* and transcriptome result by taking parameter "protein_gff, pred_gff and other_gff " according to the

Maker-P manual. The CEGMA assessment showed that our current pipeline identified 97.09 % (234 of 241) of core eukaryotic genes, while the Maker-P pipeline identified only 86.72% (209 of 241) core eukaryotic genes. The BUSCO evaluation demonstrated that 87.4 and 6.4 % of 1,440 expected plant genes were identified as completeness and fragment, respectively (Table 9). Both assessment methods suggested that for the *R. delavayi* genome our current pipeline performed better than the Maker-P pipeline.

Table 8. Summary of *R. delavayi* gene annotation

Table 9. BUSCO assessment of gene prediction comparing different pipelines

Functional annotation

Gene function annotation was assigned based on sequence and domain conservation. 1) Assignment based on sequence conservation: protein sequences of *R. delavayi* were aligned to KEGG (v76) [49], SwissProt and TrEMBL (Uniprot release 201406) [50] by BLASTP (v2.2.26) using an E-value threshold of 1e-5. Best-hit BLAST results were then used to define the gene functions. 2) Assignment based on domain conservation: InterProScan-5.11-51.0 [51] was employed to identify motifs and domains by matching against public databases Pfam [52], PRINTS [53], ProDom [54], SMART [55], and PANTHER [56]. Gene Ontology identities [57] for each gene were then obtained from the corresponding InterPro entry [58]. Overall, 85.91 % of genes were functionally annotated by at least one of the five databases above, with 22,946 InterPro entries, 16,471 GO entries, 21,210 KEGG entries, 22,693 SwissProt entries and 27,975 TrEMBL entries (Table 10).

Table 10. Statistics for functional annotations in corresponding InterPro entry

Gene Family Construction

As references, protein sequences of ten angiosperms (*Actinidia chinensis*, *Primula veris*, *Catharanthus roseus*, *Dendrobium officinale*, *Phalaenopsis equestris*, *Tarenaya hassleriana*, *Solanum tuberosum*, *Solanum lycopersicum*, *Arabidopsis thaliana* and *Oryza sativa*) were downloaded (see supporting data). For genes with alternative splicing variants, the longest transcript was selected to represent the gene. Similarities between sequence pairs were calculated using BLASTP using an E-value threshold of 1e-5. Additionally, OrthoMCL [59] was used with default parameters to identify gene family membership based on overall gene similarity combined with Markov Chain Clustering (MCL). Of all annotated genes 77.60 % were assigned to a family. A total of 14,836 families were represented, of which 1,097 were specific of *Rhododendron delavayi* (Table 11). Figure 4 showed the number of orthologous gene families shared between six flower plant genome, and there have 5,312 orthologous gene families in common with ancestral functions.

Table 11. Statistical analysis of gene families

Figure 4. Groups of orthologues shared among the angiosperms *Rhododendron delavayi* (RHOQ), *Actinidia chinensis* (KIWI), *Primula veris* (BAOC), *Catharanthus roseus* (CHAN), *Phalaenopsis equestris* (HDLH) and *Tarenaya hassleriana* (ZDIH). Venn diagram generated by http://www.interactivenn.net/.

Phylogenetic analysis

For a phylogenetic analysis 326 single copy orthologs were selected from the gene family step, and translated protein sequences were aligned in MUSCLE (v3.8.31) [60]. Next, the protein alignments were converted to corresponding coding sequences (CDS) using an in-house Perl script. Afterwards, the coding sequences of each single copy family were concatenated to form one super gene for each species. The nucleotides at position 2 (phase one site) and 3 (four fold degenerate

site) of each codon were extracted separately and were used to construct two separate phylogenetic trees in PhyML3.0 [61] specifying a HKY85 substitution model with a gamma distribution across sites. The tree using the phase one site was consistent with the tree using the four degenerate site.

Divergence time

A Bayesian relaxed molecular clock approach was used to estimate species divergence time using MCMCTREE in PAML [62] based on the four-degenerate sites data set used in phylogenetic analysis. When using previously published calibration times [63] (split of *Oryza sativa* and *Arabidopsis thaliana* fixed as 130~200 Mya), the divergence time between *R. delavayi* and *Actinidia chinensis* was estimated to be in the range of 56.1-120.8 million years ago (Figure 5).

Figure 5. Estimation of divergence time. The blue numbers on the nodes are the divergence times from present (million years ago, Mya), the red node indicates the calibrated split.

Conclusion

Now the order Ericales has three draft genome sequences of three economically important species [kiwi fruit (*Actinidia chinensis*), American cranberry (*Vaccinium macrocarpon*) and *R. delavayi*], two of which (*V.macrocarpon* and *R. delavayi*) also belongs to the family Ericaceae. The availability of the *R. delavayi* genome sequence should facilitate *de novo* genome assembly of other species in this genus, and moreover, allow scientists to investigate interactions between environmental factors and related species at a molecular level. Furthermore, phylogenetic research can now draw on a genome as resource to identify regions providing suitable resolution in this taxonomically difficult group, and it may become easier to identify genes involved in metabolite

pathways that have potential pharmaceutical importance.

Abbreviations

Gb: Gigabase; TE: Transposable element; GO: Gene Ontology; PE: pair end;

Acknowledgements

This project was supported by the Program of Science and Technology Talents Training in Yunnan province (2016HA005), the Program of Innovative Talents Promotion by the Chinese Ministry of Science and Technology (2014HE002), the Applied Basic Research Project of Yunnan Province (2016FB0858), and the National Natural Science Foundation of China (31460217, 31560225). We thank to Tobias Marczewski for his grateful help for a good language polish.

Availability of supporting data

Supporting data are available in the GigaDB database [64], and the raw data were deposited in the SRA527514 with the project accession PRJNA361437 for *Rhododendron delavayi* genome.

Actinidia chinensis: ftp://bioinfo.bti.cornell.edu/pub/kiwifruit/

Catharanthus roseus: http://bioinformatics.psb.ugent.be/orcae/overview/Catro

Primula veris: http://datadryad.org/resource/doi:10.5061/dryad.2s200

Dendrobium officinale: ftp://202.203.187.112/genome/dendrobe/

Phalaenopsis equestris: ftp://ftp.genomics.org.cn/from_BGISZ/20130120/

Solanum tuberosum: phytozome12.0 (https://phytozome.jgi.doe.gov/pz/portal.html)

Solanum lycopersicum: phytozome12.0 (https://phytozome.jgi.doe.gov/pz/portal.html)

Arabidopsis thaliana: phytozome12.0 (https://phytozome.jgi.doe.gov/pz/portal.html)

Oryza sativa: phytozome12.0 (https://phytozome.jgi.doe.gov/pz/portal.html)

Author’s contribution

LZ, JW YC, LM, and QG conceived the project. SL, FL, WX, JS, LP, and HY designed sample collection and extracted the genomic DNA. PX led the genome analysis, conducted the genome assembling, and predicted gene structure and repeat sequences. All of the authors listed above participated in discussions of the project and data. PX, LZ (Lu Zhang), QG, and JW co-drafted the manuscript, and LZ (Ling Zou), YM, and CZ helped with manuscript revision. All authors read and approved the final manuscript.

Competing interests

The authors declare that they have no competing interests.

References

1. Chamberlain D, Hyam R, Argent G, Fairweather G, Walter, KS. The genus *Rhododendron*: its classification and synonymy. Royal Botanic Garden Edinburgh. 1996.
2. Fang M., Fang R., He M., Hu L., Yang, H., Chamberlain D. Flora of China – Apiaceae through Ericaceae. Vol. 14, pp. 260–455. Science Press (China) and Missouri Botanic Garden Press (USA). 2005.
3. Gaira KS, Rawal RS, Rawat B, Bhatt ID. Impact of climate change on the flowering of *Rhododendron arboreum* in central Himalaya, India. Current Science (00113891). 2014; 106 (12).
4. Ranjitkar S., Luedeling E., Shrestha KK., Guan K., Xu J. Flowering phenology of tree *Rhododendron* along an elevation gradient in two sites in the Eastern Himalayas. International journal of biometeorology. 2013; 57(2): 225-240.
5. Bi Y, Xu J, Yang J, Li Z, Gebrekirstos A, Liang E, Yang X. Ring-widths of the above tree-line shrub *Rhododendron* reveal the change of minimum winter temperature over the past 211 years in Southwestern China. Climate Dynamics. 2016; 1-15.
6. Komac B, Esteban P, Trapero L, Caritg R. Modelization of the Current and Future Habitat Suitability of *Rhododendron ferrugineum* Using Potential Snow Accumulation. PloS one. 2016; 11(1): e0147324.
7. Cao Y, Chu Q, Ye J. Chromatographic and electrophoretic methods for pharmaceutically active compounds in *Rhododendron dauricum*. Journal of chromatography B. 2004; 812(1): 231-240.
8. Zhou W, Oh J, Li W, Kim DW, Yang MH, Jang JH, Ahn SJ, Lee HS, Ma, M. Chemical constituents of the Korean endangered species *Rhododendron brachycarpum*. Biochemical Systematics and Ecology. 2014; 56: 231-236.
9. Qiang Y, Zhou B, Gao K. Chemical constituents of plants from the genus *Rhododendron*. Chemistry & Biodiversity. 2011; 8(5): 792-815.
10. Zha HG., Milne RI, Sun H. Morphological and molecular evidence of natural hybridization between two distantly related *Rhododendron* species from the sino-himalaya. Botanical Journal of the Linnean Society, 2008; 156(1): 119-129.
11. Yu SX. Research on the problem on the problems of classification of the genus *Rhododendron*. Journal of Wuhan Botanical Research. 1986; 24(3): 161-164.
12. Zha HG, Milne RI, Sun H. Asymmetric hybridization in *Rhododendron agastum*: a hybrid taxon comprising mainly F1s in Yunnan, China. Annals of Botany. 2010; 105 (1): 89-100.
13. Ming TL, Fang RC, The phylogeny and evolution of genus *Rhododendron*, Acta Botanica Yunnanica. 1990; 12(4): 353-365.
14. Milne RI, Davies C, Prickett R, Inns LH, Chamberlain DF. Phylogeny of *Rhododendron* subgenus *Hymenanthes* based on chloroplast DNA markers: between-lineage hybridisation during adaptive radiation? Plant Systematics and Evolution. 2010; 285(3-4): 233-244.
15. Eckert AJ, Carstens BC. Does gene flow destroy phylogenetic signal? The performance of three methods for estimating species phylogenies in the presence of gene flow. Molecular Phylogenetics and Evolution. 2008; 49(3): 832-842.

16. Zha HG, Milne RI, Sun H. Morphological and molecular evidence of natural hybridization between two distantly related *Rhododendron* species from the Sino-Himalaya. Botanical Journal of the Linnean Society. 2008; 156(1): 119-129.

17. Marczewski T, Ma YP, Zhang XM, Sun WB, Marczewski AJ. Why is population information crucial for taxonomy? A case study involving a hybrid swarm and related varieties. AoB Plants. 2016; 8: plw070.

18. Fang MY, Fang RZ, He MY, Hu LZ, Yang HB, Chamberlain DF. *Rhododendron*. In: Wu ZY, Raven PH. eds. Flora of China, vol. 14. Beijing and St Louis, Science Press and Missouri Botanical Garden. 2005; 260-455.

19. Murray MG, Thompson WF. Rapid isolation of high molecular weight plant DNA. Nucleic Acids Research. 1980; 8: 4321-4325.

20. Luo R, Liu B, Xie Y, Li Z, Huang W, Yuan J, He G, Chen Y, Pan Q, Liu Y. SOAPdenovo2: an empirically improved memory-efficient short-read de novo assembler. Gigascience. 2012; 1(1):1

21. Marcais, G., and C. Kingsford. A fast, lock-free approach for efficient parallel counting of occurrences of k-mers. Bioinformatics. 2011; 27(6): 764 -770

22. Kajitani R, Toshimoto K, Noguchi H, Toyoda A, Ogura Y, Okuno M, Yabana M, Harada M, Nagayasu E, Maruyama H, et al. Efficient de novo assembly of highly heterozygous genomes from whole-genome shotgun short reads. Genome Research. 2014; 24(8): 1384-95.

23. You M., Yue Z., He W., Yang X., Yang G., Xie M., Zhan D., Baxter W., Vasseur L., Gurr M., et al. A heterozygous moth genome provides insights into herbivory and detoxification. Nature Genetics. 2013; 45(2): 220-225.

24. Grabherr MG, Haas BJ, Yassour M, Levin JZ, Thompson DA, Amit I, Adiconis A, Fan L, Raychowdhury R, Zeng Q, et al. Full-length transcriptome assembly from RNA-Seq data without a reference genome. Nat Biotechnology. 2011; 29(7): 644-52.

25. Pertea G, Huang XQ, Liang F, Antonescu V, Sultana R, Karamycheva S, Lee Y, White J, Cheung F, Parvizi B, et al. TIGR gene indices clustering tools (TGICL): a software system for fast clustering of large EST datasets. Bioinformatics. 2003; 19(5): 651-652.

26. Parra G, Bradnam K, Korf I. CEGMA: a pipeline to accurately annotate coregenes in eukaryotic genomes. Bioinformatics. 2007; 23: 1061-7.

27. Simão FA, Waterhouse RM, Ioannidis P, Kriventseva EV, Zdobnov EM. BUSCO: assessing genome assembly andannotation completeness with single copy orthologs. Bioinformatics. 2015; 31(19): 3210-3212.

28. Kent WJ. BLAT the BLAST like alignment tool. Genome Research. 2002; 12: 656-664.

29. Benson G, Tandem repeats finder: a program to analyze DNA sequence. Nucleic Acid Res. 1999; 27: 573-580.

30. Chen NS. Using RepeatMasker to identify repetitive elements in genomic sequences. Curr Protoc Bioinformatics. 2009; Chapter 4: Unit 4.10.

31. Jurka J, Kapitonov VV, Pavlicek A, Klonowski P, Kohany O, Walichiewicz J. Repbase Update, a database of eukaryotic repetitive elements. Cytogenet Genome Research. 2005; 110: 462-467.

32. Abrusán G, Grundmann N, DeMester L, Makalowski W. TEclass-a tool forautomated classification of unknown eukaryotic transposable elements. Bioinformatics. 2009; 25: 1329–30.

33. Xu Z, Wang H. LTR_FINDER: an efficient tool for the prediction of full-length LTR retrotransposons. Nucleic Acids Res. 2007; 35: W265-268.

34. Kaul S, Koo HL, Jenkins J, Rizzo M, Rooney T, Tallon LJ, Feldblyum T, Nlerman W, Benlto MI, Lin XY, et al. Analysis of the genome sequence of the flowering plant *Arabidopsis thaliana*. Nature. 2000; 408: 796-815.

35. Huang S, Ding J, Deng D, Tang W, Sun H, Liu D, Yu J. Draft genome of the *kiwifruit Actinidiachinensis*. Nature Communications. 2013; 4: 2640.

36. Qin C, Yu C, Shen Y, Fang X, Chen L, Min J, Yang Y. Whole-genome sequencing of cultivated and wild peppers provides insights into Capsicum domestication and specialization. Proceedings of the National Academy of Sciences. 2014; 111(14): 5135-5140.

37. Kelly JK, Koseva B, Mojica JP. The genomic signal of partial sweeps in *Mimulus guttatu*. Genome Biol Evol. 2013; 5(8):1457-1469

38. The Potato Genome Sequencing Consortium. Genome sequence and analysis of the tuber crop potato. Nature. 2011; 475: 189-195.

39. The Tomato Genome Consortium. The tomato genome sequence provides insights into fleshy fruit evolution. Nature. 2012; 485: 635-641

40. Altschul SF, Madden TL, Schaffer AA, Zhang JH, Zhang Z, Miller W, Lipman DJ. Gapped BLAST and PSI-BLAST: a new generation of protein database serch programs. Nucleic Acids Research. 1997; 25: 3389-3402.

41. Li, R. et al. The sequence and de novo assembly of the giant panda genome. Nature. 2010; 463: 311-317.

42. Birney E, Clamp M, Durbin R. GeneWise and Genomewise. Genome Research. 2004; 14: 988-995.

43. Stanke M, Keller O, Gunduz I, Hayes A, Waack S, Morgenstern B. AUGUSTUS: ab initio prediction of alternative transcripts. Nucleic Acid Res. 2006; 34: W435-439.

44. Burge C, Karlin S. Prediction of complete gene structures in human genomic DNA. Journal of Molecular Biology. 1997; 268: 78-94.

45. Elsik CG, Mackey AJ, Reese JT, Milshina NV, Roos DS, Weinstock GM. Creating a honey bee consensus gene set. Genome Biology. 2007; 8: R13.

46. Trapnell C, Pachter L, Salzberg SL. TopHat: discovering splice junctions with RNA-Seq. Bioinformatics. 2009; 25: 1105-1111.

47. Trapnell C, Williams BA, Pertea G, Mortazavi A, Kwan G, van Baren MJ, Salzberg SL, Wold BJ, Pachter L.Transcript assembly and quantification by RNA-Seq reveals unannotated transcriptsand isoform switching during cell differentiation. Nature Biotechnology. 2010; 28: 511–5.

48. Campbell, M.S., C. Holt, B. Moore, and M. Yandell. Genome annotation and curation using MAKER and MAKER-P. Curr. Protoc. Bioinformatics. 2014; 48: 4.11.1 –14. 11. 39.

49. Ogata H, Goto S, Sato K, Fujibuchi W, Bono H, Kanehisa M. KEGG: Kyoto Encyclopedia of Genes and Genomes. Nucleic Acids Res. 1999; 27: 29-34.

50. Bairoch A, Apweiler R. The SWISS-PROT protein sequence database and its supplement TrEMBL in 2000. Nucleic Acid Res. 2000; 28: 45-48.

51. Zdobnov EM, Apweiler R. InterProScan-an integration platform for the signature-recognition methods in InterPro. Bioinformatics. 2001; 17: 847-848.

52. Bateman A, Birney E, Durbin R, Eddy SR, Howe KL, Sconnhammer EL. The Pfam protein families database. Nucleic Acids Research. 2000; 28: 263-266.

53. Attwood TK, Cronig MD, Flower DR, Lewis AP, Madey JE, Scordis P, Selley JN, Wright W. PRINTS-S: the database formerly known as PRINTS. Nucleic Acids Res. 2000; 28: 225-227.

54. Corpet F, Gouzy J, Kahn D. Recent improvements of the ProDom database of protein domain families. Nucleic Acids Res. 1999; 27: 263-267.

55. Schult J, Copley RR, Doerks T, Ponting CP, Bork P. SMART: a web-based tool for the study of genetically mobile domains. Nucleic Acids Res. 2000; 28: 231-234.

56. Mi HY, Lazareva-Ulitsky B, Loo R, Kejariwal A, Vandergriff J, Rabkin S, Guo N, Muruganujan A, Doremieux O, Campbell MJ, et al. The PANTHER database of protein families, subfamilies, functions and pathways. Nucleic Acids Research. 2005; 33: 284-288.

57. Ashburner M, Ball CA, Blake JA, Botstein D, Butler H, Cherry JM, Davis AP, Dolinski K, Dwight SS, Eppig JT. Gene Ontology: tool for the unification of biology. Nature Genetics. 2000; 25(1): 25-9.

58. Burge S, Kelly E, Lonsdale D, Mutowo-Muellenet P, McAnulla C, Mitchell A. Manual GO annotation of predictive protein signatures: the InterPro approach to GO curation. Database. 2012; 2012: 257-264.

59. Li L, Stoeckert Jr CJ, Roos DS. OrthoMCL: identification of ortholog groups for eukaryotic genomes. Genome Res. 2003; 13(9):2178 – 89

60. Edgar RC. MUSCLE: multiple sequence alignment with high accuracy and high throughput. Nucleic Acids Res. 2004; 32(5): 1792-1797.

61. Guindon S., Dufayard J.F., Lefort V., Anisimova M., Hordijk W., Gascuel O. New Algorithms and Methods to Estimate Maximum-Likelihood Phylogenies: Assessing the Performance of PhyML 3.0. Systematic Biology. 2010; 59(3): 307-321.

62. Yang, Z. PAML 4: phylogenetic analysis by maximum likelihood. Mol Biol Evol. 2007; 24: 1586-1591.

63. Tuskan, G. A. et al. The genome of black cottonwood, Populus trichocarpa (Torr. & Gray). Science. 2006; 313: 1596-1604.

64.

Table Click here to download Table 20170529 table_TM.docx

Table 1 Sequencing libraries and data yields for whole genome shotgun sequencing

Library type	Lane	Read Length (bp)	Insert Size (bp)	Raw bases		Clean bases	
				Total bases (Gb)	Depth(X)	Total bases (Gb)	Depth(X)
PE101	2	100	170	80.47	115.30	74.12	106.20
PE151	1	150	250	59.69	85.52	47.20	67.63
PE101	4	100	500	47.89	68.62	43.58	62.44
PE101	3	100	800	42.22	60.49	36.79	52.71
MP50	2	49	2000	30.36	43.50	19.56	28.03
MP50	3	49	5000	23.11	33.11	9.06	12.98
MP50	3	49	10000	20.17	28.90	6.71	9.61
MP50	2	49	20000	19.01	27.24	4.35	6.23
MP50	1	49	40000	13.91	19.93	4.69	6.72
Total	21			336.83	482.61	246.06	352.55

Note: Sequencing depth was calculated based on a genome size of 697.94 Mb. High-quality data were obtained by filtering raw data for low-quality and duplicate reads. PE: paired-end, MP: mate pair.

Table 2 Statistics of genome size estimation by KmerFreq with k = 17, 21, 25 and 27

Genome	K-mer length(bp)	K-mer numbers	K-mer depths	Estimated Genome size	Read numbers	Genome coverage
Rhododendron delavayi	17	24,427,946,424	35	697,941,326	290,808,886	41.8×
	21	23,264,710,880	33	704,991,238	290,808,886	41.25×
	25	22,101,475,336	31	712,950,817	290,808,886	40.79×
	27	21,519,857,564	30	717,328,585	290,808,886	40.54×

Note: The genome size was estimated according to the formula: Genome size = k-mer_numbers/k-mer_depths

Table 3 Properties of the *R. delavayi* k-mer distributions for k = 25 and k =31 using jellyfish

k-mer length	k = 25	k = 31
Total k-mers	22,120,556,922	20,373,342,031
Error k-mers	615,612,427	688,273,368
Haploid coverage depth	16	14
Diploid coverage depth	31	28
Diploid genome size	693,707,887	703,038,167

Note: The genome size was estimated according to the formula: Genome size = (Total k-mers – Error k-mers)/Diploid coverage depth

Table 4 The genome assembly and completeness of *R. delavayi*

	Contig		Scaffold	
	Size(bp)	Number	Size(bp)	Number
N50	61,801	2,871	637,826	313
Minimum length	13		79	
Maximum length	581,429		3,407,404	
Total Size	657,780,215		695,092,854	
Number(>=100bp)		209,926		193,086
Number(>=2kb)		20,175		4,972
Number(>=100kb)		1,315		1,230
Number(>=1Mb)				140
CEGMA completeness				95.87% [238]
CEGMA partial				98.39% [244]
BUSCO completeness				92.8% [1337]
BUSCO fragment				1.8%% [26]

Note: Numbers of genes that match CEGMA or BUSO are shown in square brackets.

Table 5 Statistics of the assembly with different parameters

Assembler	Assembly size (bp)	Contig N50 (bp)	Scaffold N50 (bp)	K-mer (bp)	Gapcloser	Rabbit
SOAP*denovo*2	854,390,781	900	3380	63	No	No
SOAP*denovo*2	543,175,156	1,118	5,946	37	No	No
SOAP*denovo*2	1,231,272,241	19,792	67,539	87	Yes	No
SOAP*denovo*2	796,221,798	25,301	104,917	87	Yes	Yes
Platanus	750,231,563	13,232	583,084	41	No	No
Platanus	809,870,271	7,886	383,826	47	No	No
Platanus	752,607,346	54,782	584,190	41	Yes	No
Platanus	695,092,854	61,801	637,826	41	Yes	Yes

Table 6 The gene coverage of *R. delavayi* by transcriptome data

Dataset	Number	Total length (bp)	Base coverage by assembly (%)	>90% sequence in one scaffold (%)	>50% sequence in one scaffold (%)
>200bp	83,515	84,701,674	96.98	89.57	98.90
>500bp	46,582	73,471,401	96.90	85.64	99.03
>1000bp	29,816	61,377,043	96.80	82.85	99.08

Table 7 Transposable elements in the *R. delavayi* genome

	Repabse TEs	Protein TEs	*De novo* TEs	Combined TEs	
	length	length	length	length	percentage
DNA	7,882,501	7,328,645	69,812,249	77,776,557	11.19
LINE	4,811,976	12,454,813	31,065,638	36,834,088	5.30
SINE	125,792	0.00	869,547	991,785	0.14
LTR	34,884,681	52,469,776	257,040,066	260,532,496	37.48
Other	552	0.00	0.00	552	0.00
Unknown	0.00	0.00	4,565,754	4,565,754	0.67
Total	470,018,44	72,016,848	350,372,642	359,874,503	51.77

Note: Repabse TEs means RepeatMask against Repbase; Protein TEs means RepeatProteinMask result against Repbase protein; *De novo* TEs means RepeatMask against the *de novo* library; Combined TEs means the combine result of three steps.

Table 8 Summary *R. delavayi* genome annotation

Gene set		Gene numbers	Average gene length (bp)	Average CDS length (bp)	Average exon per gene	Average exon length (bp)	Average intron length (bp)
De novo	AUGUSTUS	42,672	2623.41	974.42	4.76	204.56	438.16
	GENSCAN	35,859	11242.68	1186.91	6.35	186.87	1879.03
Homolog	*A. chinensis*	45,449	3501.48	846.20	3.21	263.43	1200.29
	A. thaliana	31,950	3724.50	994.90	4.07	244.30	888.41
	C. annuum	47,672	2558.30	805.26	3.01	267.50	872.00
	M. guttatus	34,616	3454.51	963.76	3.95	244.21	845.35
	S. lycopersicum	38,800	3324.95	917.11	3.74	245.47	880.01
	S. tuberosum	39,085	2958.21	850.18	3.22	263.79	948.30
	GLEAN	29,585	4126.65	1150.32	4.84	237.78	775.53
	RNA-seq	38,273	2989.97	828.78	3.45	240.07	881.29
	Final set	32,938	4434.22	1153.21	4.62	249.70	785.08

Table 9. BUSCO assessment of gene prediction with different pipelines

BUSCO benchmark	Current pipeline		Maker-P	
	Number	Percentage	Number	Percentage
Total BUSCO groups searched	1440		1440	
Complete single-copy BUSCOs	1188	82.5	1056	73.3
Complete duplicated BUSCOs	70	4.9	67	4.7
Fragmented BUSCOs	92	6.4	152	10.6
Missing BUSCOs	90	6.2	165	11.4

Table 10 Statistics for functional annotations in corresponding InterPRro entry

	Numbers of matching genes	Percent of annotated genes (%)
InterPro	22,946	69.66
GO	16,471	50.00
KEGG	21,210	64.39
Swissprot	22,693	68.90
TrEMBL	27,975	84.93
Annotated	28,296	85.91

Table 11 Statistic analysis of gene families

Species	Number of genes	Genes in families	Unclustered genes	Number of families	Unique families	Average number of genes per family
R. delavayi	32,938	25,560	7,378	14,836	1097	1.72
A. chinensis	39,040	26,061	12,979	14,047	1100	1.86
P. veris	18,269	15,080	3,189	11,434	180	1.32
C. roseus	28,172	15,122	13,050	10,725	1231	1.41
D. officinale	35,474	25,525	9,949	14,416	1091	1.77
P. equestris	29,413	21,086	8,327	13,834	705	1.52
T. hassleriana	39,881	38,100	1,781	14,399	623	2.65
S. tuberosum	34,879	28,093	6,786	16,118	667	1.74
S. lycopersicum	33,585	25,623	7,962	17,139	532	1.50
A. thaliana	26,637	23,007	3,630	14,482	539	1.59
O. sativa	38,942	26,644	12,298	13,632	2020	1.95

Figure 1

Click here to download Figure Figure 1.tif

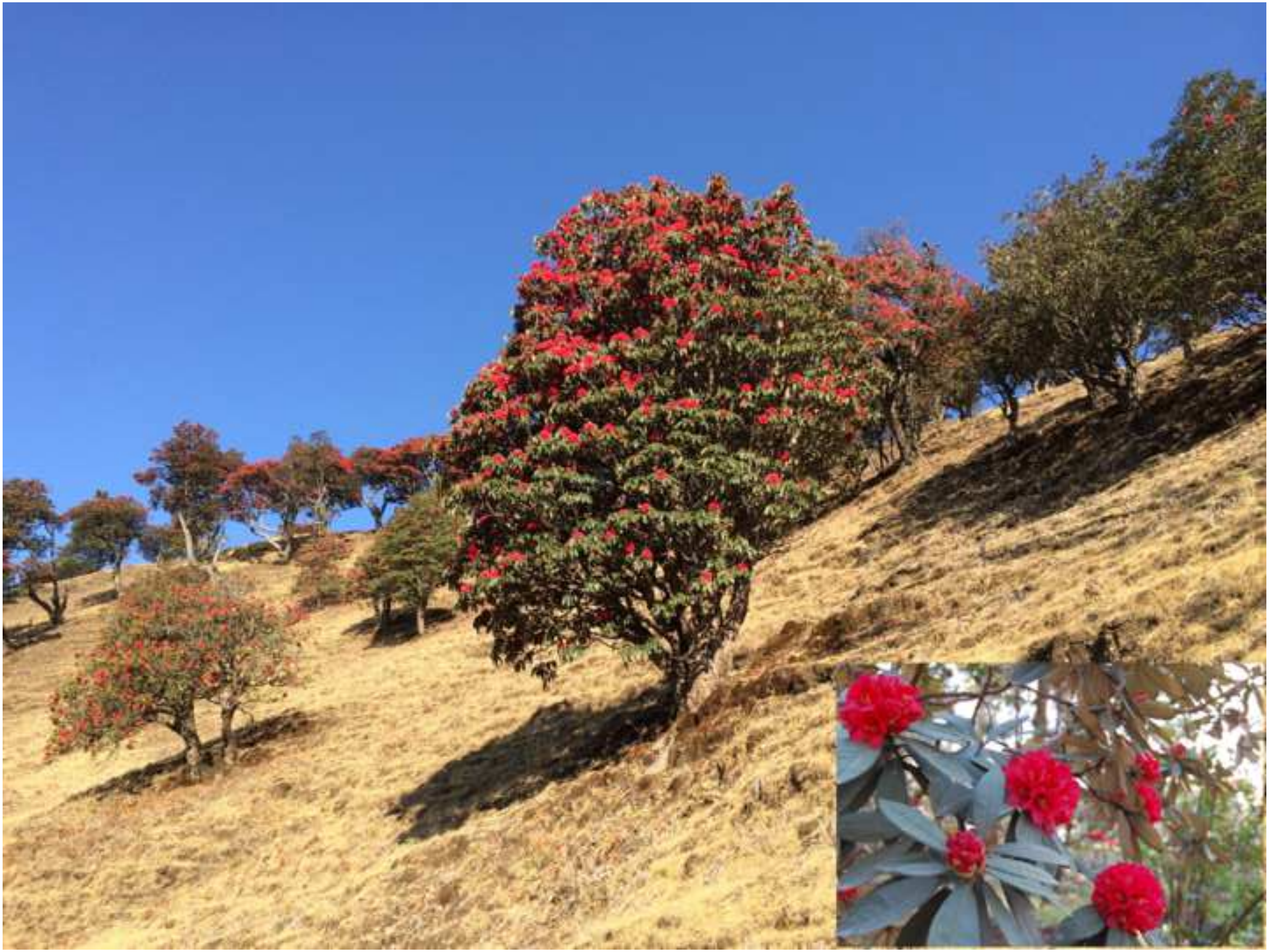

Figure 2

Click here to download Figure Figure 2.pdf

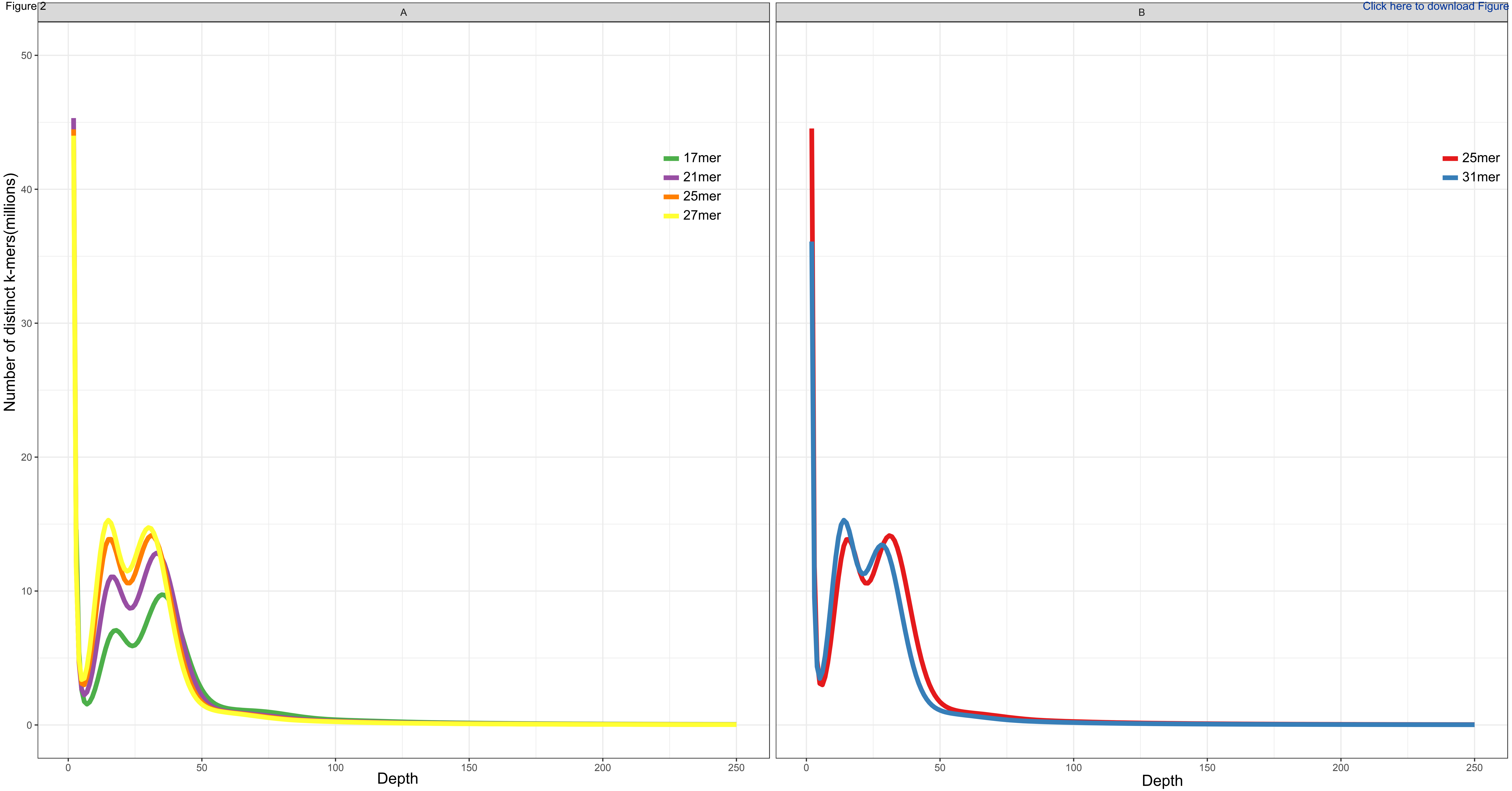

Figure 3 Click here to download Figure Figure 3.pdf

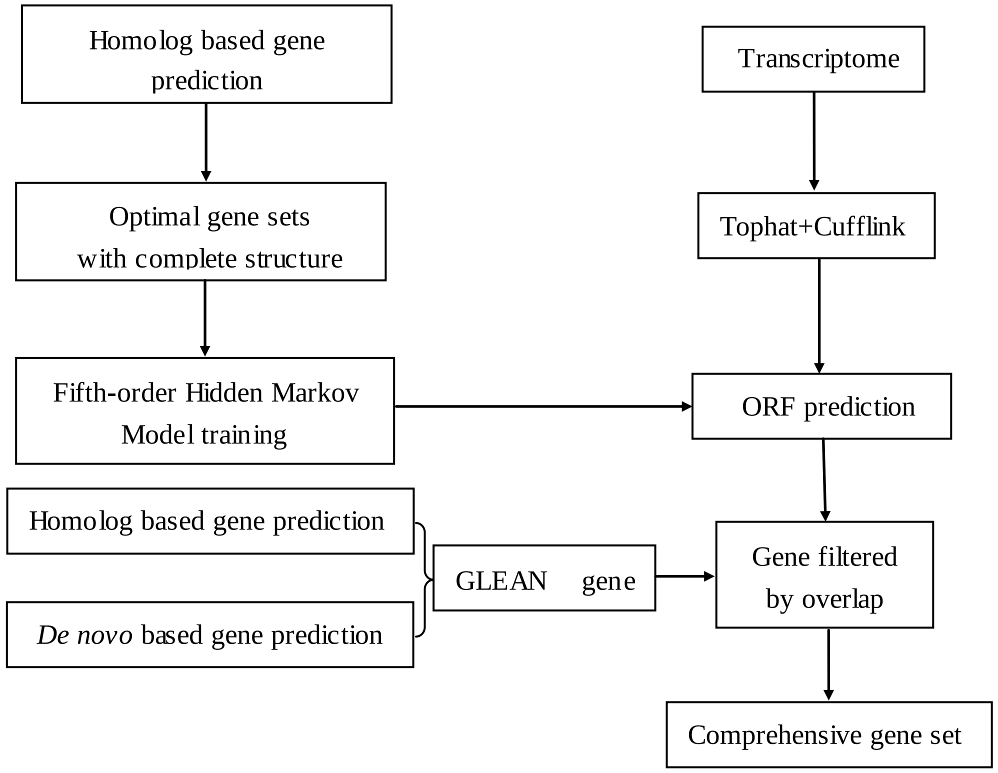

Figure 4

Click here to download Figure Figure 4.pdf

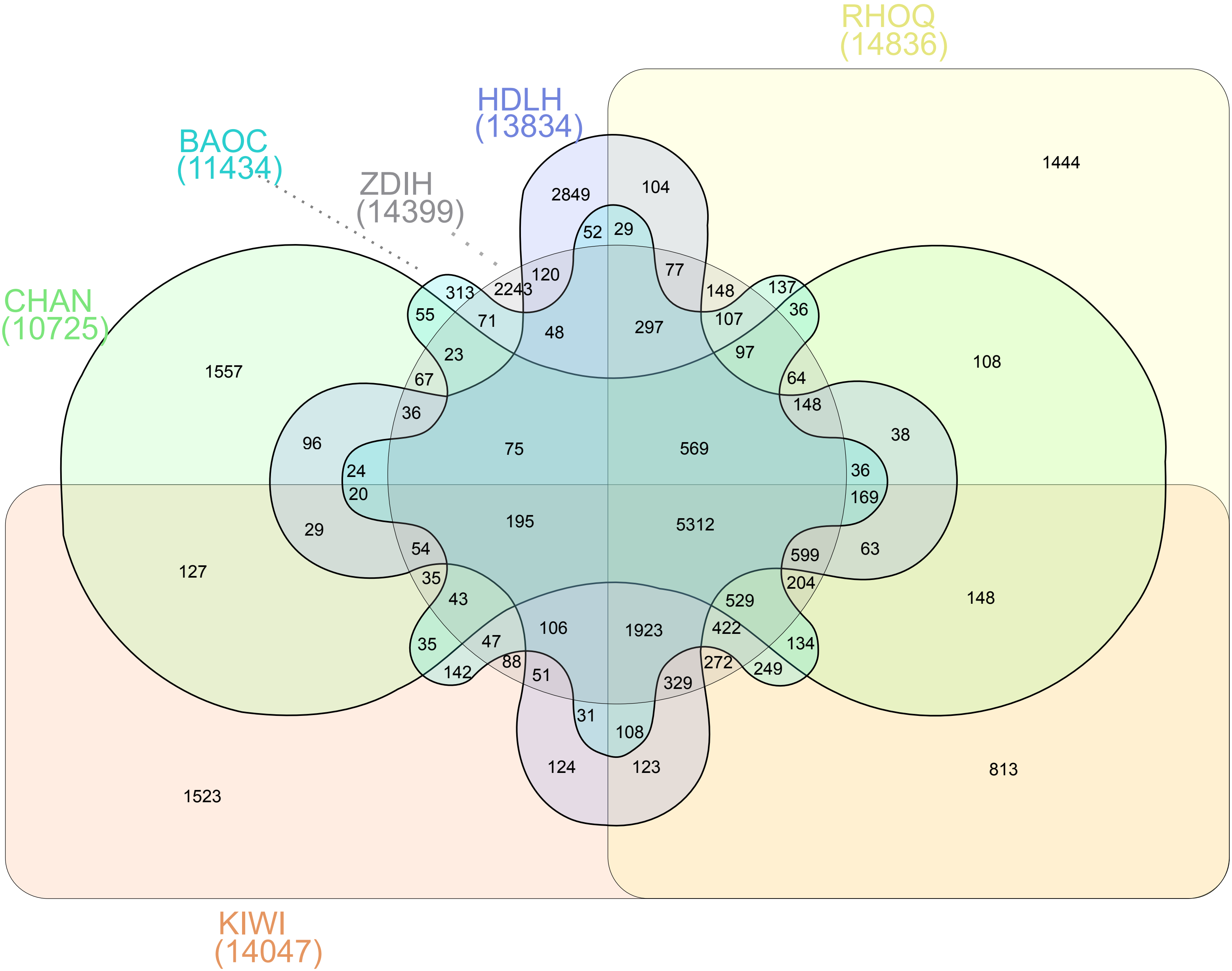

Figure 5

Click here to download Figure Figure 5.pdf

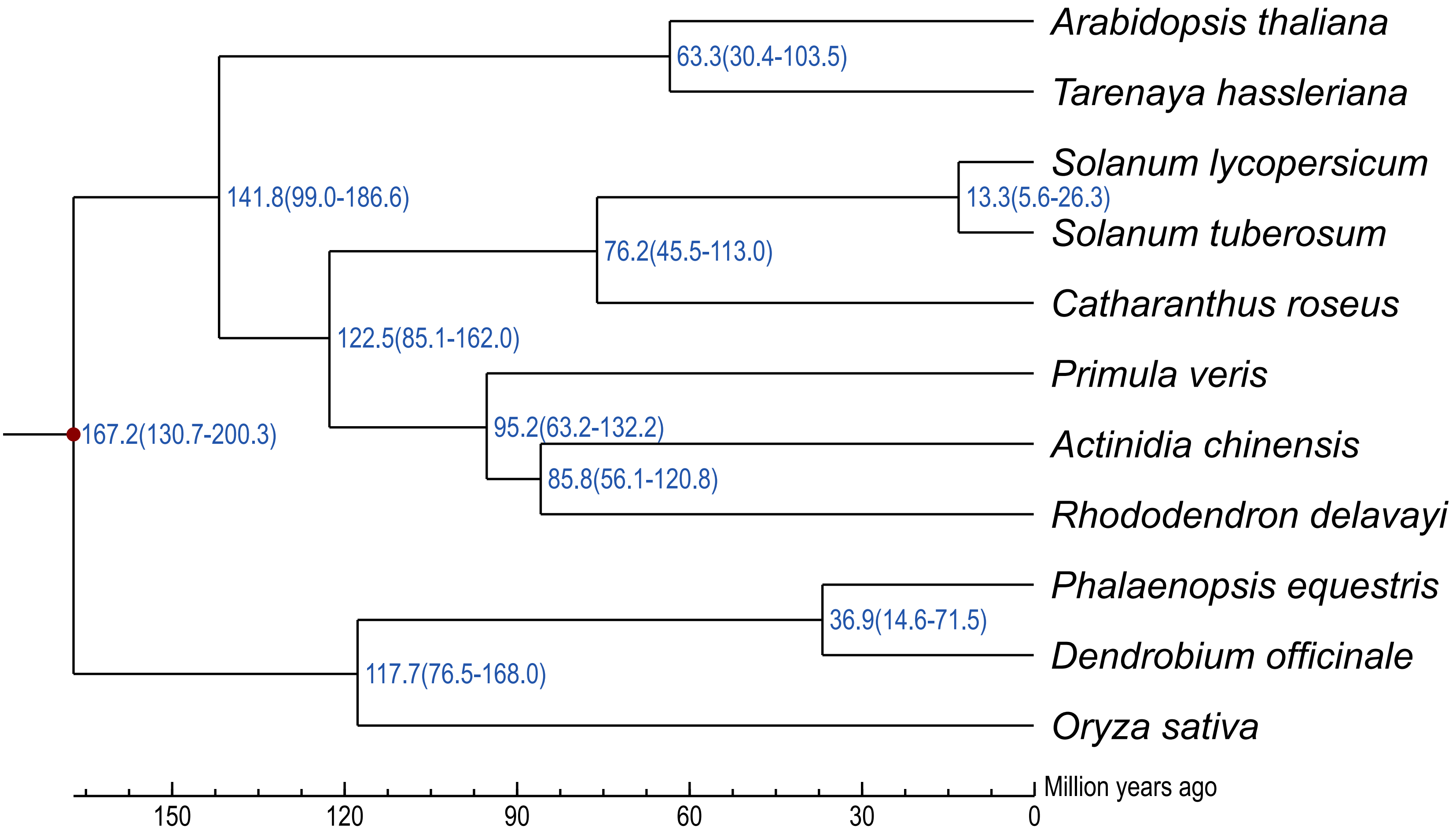

Supplement: GIGA-D-17-00027_Revision-1.pdf [file gix076_GIGA-D-17-00027_Revision-1.pdf]
