# Supplementary material for: The draft genome assembly of Rhododendron delavayi Franch. var. delavayi
Source: Gigascience. 2017 Aug 26;6(10):1–11. doi: 10.1093/gigascience/gix076 (PMC5632301; doi:10.1093/gigascience/gix076)
Supplement: Response-to-Reviewer-Comments_Original-Submission.pdf [file gix076_Response-to-Reviewer-Comments_Original-Submission.pdf]

Author's Response To Reviewer Comments

Dear the Editor,
Thank you very much for returning our manuscript (GIGA-D-17-00027) entitled “The draft genome assembly of Rhododendron delavayi Franch. var. delavayi” along with some constructive comments and suggestions from three referees. We are very grateful for their efforts.
We have done our best to respond positively to all comments of our paper. We include a file below going through these comments one by one.
We resubmit here the revised manuscript and we hope this version is now suitable for the publication in GigaScience. If you have any further questions or requirements, please do not hesitate to contact the corresponding author.

Yours sincerely,
Jihua Wang

Reviewer reports:
Reviewer #1:
Summary of the key results
The manuscript titled "The draft genome assembly of Rhododendron delavayi Franch." describes the genome sequencing, assembling and annotation of the species Rhodorendron delavayi. The authors present the stats for the assembly (total size of 695 Mb) and the annotation (32,928 genes).
Overall evaluation
The assembly and the annotation presented in the manuscript looks correct and it may be an important piece of information to understand the genome evolution in the Ericaceae family, but the authors should use different tools (e.g. SOAPdenovo2 and/or ALLPath-LG for the assembly; MAKER-P for the annotation) to have a stronger support the quality of the work. The tool used to assess/improve the quality of the assembly (Rabbit) is not publicly available so it is impossible to evaluate how good or bad is. Additionally there is an important lack of deliverable products such as WGD and gene families. In general, the authors should complete the analysis with those and any other genomic analysis that can help to appreciate how different (or similar) is the Rhododendron genome to the previously published genomes. Finally, the authors should check carefully the text, tables and figures looking for mistakes (e.g. Table 5).
Major concerns
* Data description (Page 6, line 115): Authors used Platanus for the genome assembly. I'll recommend to use at least one other assemblers to compare the results (e.g. SOAPdenovo and/or ALLPath-LG).
Response:
Because of the high heterozygosity of Rhododendron delavayi, we have also tried to use SOAPdenovo to assemble the genome, but the results of this assembly were not as good as those from Platanus (e. g. both the contig/scaffold N50 and the unigene coverages). Moreover the original size of the genome assembled by SOAPdenovo is twice as large as the genome size inferred by Platanus (Table 5).

* Data description (Page 6, line 129): If no article describing the program used to assess/optimize the assembly (Rabbit), the authors should at least write a paragraph describing the possible results. Additionally the program should be publicly available to test it. Does this program map the reads back to the assembly and filter them based in the coverage? If so, which coverage was used? Does this program assess the weakly supported scaffolds? Does this program breaks the weak joints?
Response:
The program Rabbit had been used in the genome assembly of Plutella xylostella that was published in 2013 (http://www.nature.com/ng/journal/v45/n2/abs/ng.2524.html). The software Rabbit was available from ftp://ftp.genomics.org.cn/pub/Plutellaxylostella/Rabbit_linux-2.6.18-194.blc.tar.gz. In Rabbit, The Poisson-based K-mer model was used to determine the heterozygosity in the genome repeat sequences with K-mers at low frequency and discarding K-mers at high frequency.
In this program, we first constructed a k-mer frequency table based on the reads from the small insert libraries using Jellyfish, and then constructed a k-mer frequency table using the same k-mer with Jellyfish based on the scaffolds by Rabbit. In that case, the k-mer frequencies that were unique or shared to scaffolds would be defined. If the percentage of shared k-mer/unique k-mer in a scaffold exceeds a threshold, this scaffold would be removed. So we just use the unique or shared k-mer frequencies but not the depth or coverage information.

* The manuscript could be summarized as, we sequence this genome, assembly it and annotate it, with these stats, but the analysis of the genome is missing. The authors should perform several common analysis frequently used in the genome articles, for example: 1- An ancestral whole genome duplication analysis based in the Synonymous substitution ration for pairs of homologs; 2- Gene family analysis comparing plant models (e.g. Arabidopsis, rice, grape, tomato) and closed related species (e.g. kiwi) with the sequenced species, focusing some of the analysis in gene families that could be expanded in the Rhododendron genus related with the ecology/evolutionary history of this species/genus.
Response:
The gene family have been constructed and analyzed among six flower ornamental plants (Actinidia chinensis, Primula veris, Catharanthus roseus, Dendrobium officinale, Phalaenopsis equestris, Tarenaya hassleriana), and the divergence time were also calculated between Rhododendron delavayi and Actinidia chinensis. As we planned to firstly submit this manuscript as a Data Note, and subsequent studies involved in population genomics and hybrid speciation issue relating to R. delavayi are now undergoing.

Minor concerns
* Data description (Page 6, line 66): Authors should describe how they obtained the DNA and RNA (e.g. tissue…) used for the sequencings.
Response:
We have added corresponding descriptions in the revised text (Page 4, lines 80 -85).

* Table 1: Depth(X) column data is missing. Probably the long fragments libraries (e.g. >2000 bp for insert size) should be named as MP (Mate Pairs) instead PE.
Response:
That is our mistake. Now, the data for sequence depth has been added in the last column, and the

long fragments libraries (e.g. >2000 bp for insert size) have been named as MP (Mate Pairs) instead of PE in Table 1.

* Data description (Page 8, line 108): Probably is not necessary to describe in such detail the method and its application of how was calculated the genome size. Nevertheless it could be useful to use tools such as GenomeScope (http://qb.cshl.edu/genomescope/) to supply an estimation in the heterozygosity of the sample. Additionally I would recommend to estimate the genome size with other kmers (e.g. 25 and 31) for a better estimation.
Response:
We have separately estimated the genome size in KmerFreq with four kmers (17, 21, 25, 27), and in jellyfish with two kmers (25, 31). After this, we delivered the histogram result from jellyfish to GenomeScope to estimate the heterozygosity (Page 6 - 7). The related results are given in Table 2 and table 3.

* Probably the assembly stats will benefit from other stats such as: Minimum contig/scaffold size, longest contig/scaffold, contigs/scaffolds > 100 Kb, contigs/scaffolds > 1 Mb.
Response:
Such information was added to Table 4.

* Table 5 should contain more detail about the repeats. For example, specifying repeat types such as Gypsy or Copia for the LTRs.
Response:
We added Gypsy and Copia subtype ratios in the text (page 11, line 220).

* Table 6: Numbers? Does this column represent gene number? If so, there is a mistake and the numbers should be in the order of ten thousands. Please check and change the header of the table accordingly.
Response:
The typing mistakes have been corrected, and the table header of table 8 has been changed as suggested.

* Probably the use of Maker-P for the genome annotation will improve the annotation.
Response:
We did try using Maker-P to predict the gene model, but the BUSCO evaluation was not as good as the current version (Page 12, line 262). Our current prediction version seems better and the prediction quality is enough for subsequent analysis.

* Stats such as number of genes per scaffold will help to understand how useful could be the genome to study microsynteny.
Response:
The number of genes per scaffold has been stated in the revised text (line 260).

Discretionary suggestions
* Abstract (Page 1, Line 12): Findings should summarize the results. The amount of reads produced could be simplified saying a total of "A total of 336.83 Gb of raw data were generated by Illumina 2000".

Response:
Rewritten it as suggested.

* Background (Page 2, line 33): Check the number of species. Rhododendron is a genus that has been described with 2,144 species names from which 641 has been accepted. Some literature sources describe ~1,100 species. The assignment of ~1,000 species for a region looks inaccurate.
Response:
In total over 1000 species of Rhododendron were recognized all over the world. There are 567 species in 6 subgenera in China, of these approx. 80% are endemic (Fang et al., 2005).

Fang, M., Fang, R., He, M., Hu, L., Yang, H. & Chamberlain, D. (2005) Flora of China – Apiaceae through Ericaceae. Vol. 14, pp. 260–455. Science Press (China) and Missouri Botanic Garden Press (USA)

* Background (Page 3, line 42): Probably a small paragraph extending the taxonomic classification of the species used in this study will be beneficial for the reader.
Response:
Taxonomically, four varieties have been previously identified in this species, Rhododendron delavayi var. peramoenum with narrow leaves and reported from western Yunnan, NE India and Myanmar whereas R. delavayi var. delavayi with relatively broad leaves than former and shows extensive distribution throughout the range in China. Another two varieties R. delavayi var. adenostylum and R. delavayi var. pilostylum were recently proved to be hybrids between pure R. delavayi and R.irroratum (Marczewski et al., 2016).

Marczewski T, Ma Y P, Zhang X M, et al. Why is population information crucial for taxonomy? A case study involving a hybrid swarm and related varieties. AoB Plants, 2016, 8: plw070.
* Data description (Page 7, line 133): Misstype of "minimum" as "mini" in the description of the parameters.
Response:
Has been changed (Page 9, line 188).

Reviewer #2:
The manuscript describes de novo assembly of the R. delavayi genome, an ornamental plant with medicinal potential.
There are several issues that need to be addressed by the authors regarding the genome assembly, and language corrections + references
* The biggest issue is with the genome assembly using kmer = 17 (line #99 and Table 2). There should be stats from a number of assemblies using different kmers to show what was the best kmer for this genome. Table 2 has stats only for 1 kmer (17). It would also be informative to add the peaks obtained at different Kmers (Figure 2). There is no explanation why this kmer was selected as optimal for this genome assembly.
Response:
We are sorry that the text suggested that the genome assembly only used kmer = 17. This was not the case, and different k-mers were tested to assemble the genome by kmer analysis, whereas the genome size was estimated just using k-mer=17. Now we added four k-mers (17, 21, 25 and 27) for KmerFreq and two k-mers (25 and 31) for jellyfish to do k-mer analysis, furthermore, we

added descriptions to the peaks obtained at different k-mers.

* Please go over the tables and make sure the numbers are correct. For example, in Table 6 the 'Numbers' column is missing the last digits
Response:
We have carefully checked the text and made corresponding corrections to avoid any mistakes.

* Please make the table column headers more informative , for example in table 7 instead of "Numbers" and "Percent" use "Number of matching genes" and "percent of annotated genes". More verbose table captions would also help.
Response:
Changed these table column headers as suggested.

* Line #212: "Fig. 2" should be "Fig. 3"
Response:
Has been changed in line 226.

* In the 'Conclusions' (line #28, and #224) the authors mention breeding of elite varieties. Are there any breeding programs of plants in this family/genus? The R. delavayi genome has scientific significance, but still a long way to be applied in a breeding program.
Response:
Yes, there have many studies about molecular breeding programs of plants in this family or genus, e.g. QTL analysis (Dunemann et al., 1999 Keyser et al., 2010; Keyser et al., 2013), transcriptome analysis (Dhanaraj et al., 2007; Rowland et al., 2012; Zifkin et al., 2012; Die et al., 2013) to find many functional genes probably applied in molecular breeding such as flowering time, flower color, leaf characteristics, cold acclimation, flavonoid metabolism and so on. On the other hand, genetic transformation has been successfully established in some Rhododendron cultivars and species (Pavingerová et al., 1997; Mertens et al., 1998; Knapp et al., 2001). The publication of the R. delavayi genome would facilitate locating genes or loci accounting for ornamental traits, environmental adaptation, pharmaceutically important compounds and so on, and accelerate molecular breeding to release elite varieties.
Dunemann F, Kahnau R, Stange I. Analysis of complex leaf and flower characters in Rhododendron using a molecular linkage map [J]. TAG Theoretical and Applied Genetics, 1999, 98(6): 1146-1155.
De Keyser E, Shu Q Y, Van Bockstaele E, et al. Multipoint-likelihood maximization mapping on 4 segregating populations to achieve an integrated framework map for QTL analysis in pot azalea (Rhododendron simsii hybrids) [J]. BMC molecular biology, 2010, 11(1): 1.
De Keyser E, Lootens P, Van Bockstaele E, et al. Image analysis for QTL mapping of flower colour and leaf characteristics in pot azalea (Rhododendron simsii hybrids) [J]. Euphytica, 2013, 189(3): 445-460.
Dhanaraj A L, Alkharouf N W, Beard H S, et al. Major differences observed in transcript profiles of blueberry during cold acclimation under field and cold room conditions [J]. Planta, 2007, 225(3): 735-751.
Rowland L J, Alkharouf N, Darwish O, et al. Generation and analysis of blueberry transcriptome sequences from leaves, developing fruit, and flower buds from cold acclimation through deacclimation [J]. BMC plant biology, 2012, 12(1): 46.

Rowland L J, Alkharouf N, Darwish O, et al. Generation and analysis of blueberry transcriptome sequences from leaves, developing fruit, and flower buds from cold acclimation through deacclimation [J]. BMC plant biology, 2012, 12(1): 46.
Die J V, Rowland L J. Elucidating cold acclimation pathway in blueberry by transcriptome profiling [J]. Environmental and experimental botany, 2014, 106: 87-98.
Pavingerová D, Bríza J, Kodýtek K, et al. Transformation of Rhododendron spp. using Agrobacterium tumefaciens with a GUS-intron chimeric gene [J]. Plant science, 1997, 122(2): 165-171.
Mertens M, Heursel J, Van Bockstaele E, et al. Inheritance of foreign genes in transgenic azalea plants generated by Agrobacterium-mediated transformation[C]//XXV International Horticultural Congress, Part 11: Application of Biotechnology and Molecular Biology and Breeding-Gene 521. 1998: 127-132.
Knapp J, Kausch A, Auer C, et al. Transformation of Rhododendron through microprojectile bombardment [J]. Plant Cell Reports, 2001, 20(8): 749-754.
* The references do not match with the text starting #15, please go over the references and make sure they align with the text (e.g. the reference in the text to SOAP [16] , and later to the tomato genome [35] and so on)
Response:
We have gone over the references and made corrections.

* Please go over the language, especially starting with the 'Sequence Assembly' section. There are many sentences with passive voice, and sentences that are not very clear (e.g. lines 116- 136, the 'Genome Evaluation' section)
Response:
The text has been revised by a scientific researcher who is fluent in English, and we will express our thanks for his help in ‘Acknowledgement’ part.

* Page 3 line #58 "...reduced connectivity of plants ...." - this is not clear, please rephrase
Response:
Has been rephrased on Page 4, line 69.

* Page 5 line #100 "to estimate the Ma Ying azalea..." is this the common name or a variety name? I have not seen this name mentioned elsewhere in the paper.
Response:
This name has been changed in Line 127.

* The genome was submitted to SRA. Where can the functional annotation be accessed?
Response:
Now the functional annotation is deposited in the temp ftp site of gigascience: ftp://user20@climb.genomics.cn with the available username ‘user20’ and corresponding password ‘WangJihua’. Once the manuscript is successfully accepted by the journal, all these data will be moved to GigaDB with accession numbers.

Reviewer #3:
The manuscript describes the sequencing and assembly of the ornamental plant Rhododendron delavayi. The manuscript was submitted as a "Data Note" and the manuscript is focused

appropriately on the generation of the assembly, genome annotation and data access.
Overall the manuscript adequately describes the sequencing, assembly, annotation for R. delavayi. The manuscript has several passages (mostly in the Introduction) where the phrasing is odd or there are grammar mistakes and it should be revised before being published.

Minor Comments:
* The versions for all the software used should be stated.
Response:
Added corresponding descriptions as suggested.

* Line 45 - high-through-put sequencing -> genome sequencing
Response:
This sentence has been deleted.

* Line 49 - research on bioinformatics -> genomics (or functional genomics)
Response:
Rewritten this sentence in Line 76.

* Line 55 - morphology -> appearance
Response:
Rewritten this sentence in Line 67 as suggested.

* Lines 74-77 - odd phrasing to describe the mate-pair library protocol
Response:
Rewritten Lines 88-100.

* Line 100 - Ma Ying azalea - single use of this name in the manuscript. It's not consistent and potentially confusing.
Response:
Made correction in Line 127.

* Line 120 -"proper parameters were chosen"
Response:
Made correction in Line 155.

* Line 171 - typo - Solanum tuberosum
Response:
Made correction in Line 229.

* Line 196 - typo – GLEAN
Response:
Made correction in Line 255.

* Table 6 - Numbers column missing the final digit
Response:
The format of Table 8 has been adjusted.
