# Supplementary material for: The draft genome assembly of Rhododendron delavayi Franch. var. delavayi
Source: Gigascience. 2017 Aug 26;6(10):1–11. doi: 10.1093/gigascience/gix076 (PMC5632301; doi:10.1093/gigascience/gix076)
Supplement: Response-to-Reviewer-Comments_Revision-1.pdf [file gix076_Response-to-Reviewer-Comments_Revision-1.pdf]

Author's Response To Reviewer Comments

Dear the Editor,
Thank you very much for returning our manuscript (GIGA-D-17-00027) entitled “The draft genome assembly of Rhododendron delavayi Franch. var. delavayi” once again along with some suggestions from the Reviewer #2. We are very grateful for this efforts.
We have done our best to respond positively to all comments of our paper. We include a file below going through these comments one by one.
We resubmit here the revised manuscript and we hope this version is now suitable for the publication in GigaScience. If you have any further questions or requirements, please do not hesitate to contact the corresponding author.

Yours sincerely,
Jihua Wang

Reviewer reports:
Reviewer #2: Lines 53-57 : please go over the paragraph and make it more clear - it is not clear if this paragraph is talking about the ease of hybridization or something with some subgenus taxa

Response: We have resentenced this paragraph and make it more clear in lines 51-52.

Lines 72-73 : consider removing the words 'to facilitate future research' . The next sentence describes what is the expected outcome of the genome sequence
Response: We have deleted the words 'to facilitate future research' as suggested.

Table 6 - Does the column 'number' refer to number of genes?
Response: Yes, we have rephrased it and change the header of the table accordingly.

Table 5 - the text points to table 4 (line 199) and then table 6 (line 204) , and no reference in the text to table 5
Response: Please see the text points to table 5 (line 182-183).

Gene annotation - please add number of unique genes in the species, that did not have any match to other known genes
Response: Now we have added the number of matching genes that unannotated and its corresponding percent in the last row of Table 10.

Phylogenetic analysis - a figure with the trees would be helpful
Response: We have added corresponding figures for the trees in figure 5.

I could not log into the authors' ftp site with the user and password stated in the response to reviewers.
Response: At this before, we had uploaded all related files to the temporary FTP server, but now we also could not log into the ftp site (ftp://user20@climb.genomics.cn) with the user name (user20) and password (WangJihua). We do not know what happened to these data, and we still

await for your curators's instructions.
