# Supplementary material for: The draft genome assembly of Rhododendron delavayi Franch. var. delavayi
Source: Gigascience. 2017 Aug 26;6(10):1–11. doi: 10.1093/gigascience/gix076 (PMC5632301; doi:10.1093/gigascience/gix076)
Supplement: Reviewer-1-Report-(Original-Submission).pdf [file gix076_Reviewer-1-Report-(Original-Submission).pdf]

Reviewer Report

Title: The draft genome assembly of Rhododendron delavayi Franch. var. delavayi

Version: Original Submission **Date:** 3/5/2017

Reviewer name: Aureliano Bombarely Gomez

Reviewer Comments to Author:

Summary of the key resultsThe manuscript titled "The draft genome assembly of Rhododendron delavayi Franch" describes the genome sequencing, assembling and annotation of the species Rhodorendron delavayi. The authors present the stats for the assembly (total size of 695 Mb) and the annotation (32,928 genes).Overall evaluationThe assembly and the annotation presented in the manuscript looks correct and it may be an important piece of information to understand the genome evolution in the Ericaceae family, but the authors should use different tools (e.g. SOAPdenovo2 and/or ALLPath-LG for the assembly; MAKER-P for the annotation) to have a stronger support the quality of the work. The tool used to assess/improve the quality of the assembly (Rabbit) is not publicly available so it is impossible to evaluate how good or bad is. Additionally there is an important lack of deliverable products such as WGD and gene families. In general, the authors should complete the analysis with those and any other genomic analysis that can help to appreciate how different (or similar) is the Rhododendron genome to the previously published genomes. Finally, the authors should check carefully the text, tables and figures looking for mistakes (e.g. Table 5).Major concerns* Data description (Page 6, line 115): Authors used Platanus for the genome assembly. I'll recommend to use at least one other assemblers to compare the results (e.g. SOAPdenovo and/or ALLPath-LG).* Data description (Page 6, line 129): If no article describing the program used to assess/optimize the assembly (Rabbit), the authors should at least write a paragraph describing the possible results. Additionally the program should be publicly available to test it. Does this program map the reads back to the assembly and filter them based in the coverage? If so, which coverage was used? Does this program assess the weakly supported scaffolds? Does this program breaks the weak joints?* The manuscript could be summarized as, we sequence this genome, assembly it and annotate it, with these stats, but the analysis of the genome is missing. The authors should perform several common analysis frequently used in the genome articles, for example: 1- An ancestral whole genome duplication analysis based in the Synonymous substitution ration for pairs of homologs; 2- Gene family analysis comparing plant models (e.g. Arabidopsis, rice, grape, tomato) and closed related species (e.g. kiwi) with the sequenced species, focusing some of the analysis in gene families that could be expanded in the Rhododendron genus related with the ecology/evolutionary history of this species/genus.Minor concerns* Data description (Page 6, line 66): Authors should describe how they obtained the DNA and RNA (e.g. tissue…) used for the sequencings.* Table 1: Depth(X) column data is missing. Probably the long fragments libraries (e.g. >2000 bp for insert size) should be named as MP (Mate Pairs) instead PE.* Data description (Page 8, line 108): Probably is not necessary to describe in such detail the method and its application of how was calculated the genome size. Nevertheless it could be useful to use tools such as GenomeScope (http://qb.cshl.edu/genomescope/) to supply an estimation in the heterozygosity of the sample.

Additionally I would recommend to estimate the genome size with other kmers (e.g. 25 and 31) for a better estimation.* Probably the assembly stats will benefit from other stats such as: Minimum contig/scaffold size, longest contig/scaffold, contigs/scaffolds > 100 Kb, contigs/scaffolds > 1 Mb.* Table 5 should contain more detail about the repeats. For example, specifying repeat types such as Gypsy or Copia for the LTRs.* Table 6: Numbers? Does this column represent gene number? If so, there is a mistake and the numbers should be in the order of ten thousands. Please check and change the header of the table accordingly.* Probably the use of Maker-P for the genome annotation will improve the annotation.* Stats such as number of genes per scaffold will help to understand how useful could be the genome to study microsynteny.Discretionary suggestions* Abstract (Page 1, Line 12): Findings should summarize the results. The amount of reads produced could be simplified saying a total of "A total of 336.83 Gb of raw data were generated by Illumina 2000".* Background (Page 2, line 33): Check the number of species. Rhododendron is a genus that has been described with 2,144 species names from which 641 has been accepted. Some literature sources describe ~1,100 species. The assignment of ~1,000 species for a region looks inaccurate.* Background (Page 3, line 42): Probably a small paragraph extending the taxonomic classification of the species used in this study will be beneficial for the reader.* Data description (Page 7, line 133): Misstype of "minimum" as "mini" in the description of the parameters

Level of Interest

Please indicate how interesting you found the manuscript: An article whose findings are important to those with closely related research interests

Quality of Written English

Please indicate the quality of language in the manuscript: Needs some language corrections before being published

Declaration of Competing Interests

Please complete a declaration of competing interests, considering the following questions:

- Have you in the past five years received reimbursements, fees, funding, or salary from an organisation that may in any way gain or lose financially from the publication of this manuscript, either now or in the future?
- Do you hold any stocks or shares in an organisation that may in any way gain or lose financially from the publication of this manuscript, either now or in the future?
- Do you hold or are you currently applying for any patents relating to the content of the manuscript?
- Have you received reimbursements, fees, funding, or salary from an organization that holds or has applied for patents relating to the content of the manuscript?
- Do you have any other financial competing interests?

- Do you have any non-financial competing interests in relation to this paper?

If you can answer no to all of the above, write 'I declare that I have no competing interests' below. If your reply is yes to any, please give details below.

I declare that I have no competing interests

I agree to the open peer review policy of the journal. I understand that my name will be included on my report to the authors and, if the manuscript is accepted for publication, my named report including any attachments I upload will be posted on the website along with the authors' responses. I agree for my report to be made available under an Open Access Creative Commons CC-BY license (http://creativecommons.org/licenses/by/4.0/). I understand that any comments which I do not wish to be included in my named report can be included as confidential comments to the editors, which will not be published.

I agree to the open peer review policy of the journal
