# Supplementary material for: The draft genome assembly of Rhododendron delavayi Franch. var. delavayi
Source: Gigascience. 2017 Aug 26;6(10):1–11. doi: 10.1093/gigascience/gix076 (PMC5632301; doi:10.1093/gigascience/gix076)
Supplement: Reviewer-2-Report-(Original-Submission).pdf [file gix076_Reviewer-2-Report-(Original-Submission).pdf]

Reviewer Report

Title: The draft genome assembly of Rhododendron delavayi Franch. var. delavayi

Version: Original Submission **Date:** 3/9/2017

Reviewer name: Naama Menda

Reviewer Comments to Author:

The manuscript describes de novo assembly of the R. delavayi genome, an ornamental plant with medicinal potential.There are several issues that need to be addressed by the authors regarding the genome assembly, and language corrections + references* The biggest issue is with the genome assembly using kmer = 17 (line #99 and Table 2) . There should be stats from a number of assemblies using different kmers to show what was the best kmer for this genome. Table 2 has stats only for 1 kmer (17) . It would also be informative to add the peaks obtained at different Kmers (Figure 2) . There is no explanation why this kmer was selected as optimal for this genome assembly* Please go over the tables and make sure the numbers are correct. For example, in Table 6 the 'Numbers' column is missing the last digits* Please make the table column headers more informative , for example in table 7 instead of "Numbers" and "Percent" use "Number of matching genes" and "percent of annotated genes". More verbose table captions would also help.* Line #212: "Fig. 2" should be "Fig. 3"* In the 'Conclusions' (line #28 , and #224) the authors mention breeding of elite varieties. Are there any breeding programs of plants in this family/genus? The R. delavayi genome has scientific significance , but still a long way to be applied in a breeding program* The references do not match with the text starting #15, please go over the references and make sure they align with the text (e.g. the reference in the text to SOAP [16] , and later to the tomato genome [35] and so on)* Please go over the language, especially starting with the 'Sequence Assembly' section. There are many sentences with passive voice, and sentences that are not very clear (e.g. lines 116- 136, the 'Genome Evaluation' section)* Page 3 line #58 "...reduced connectivity of plants ...." - this is not clear, please rephrase* Page 5 line #100 "to estimate the Ma Ying azalea..." is this the common name or a variety name? I have not seen this name mentioned elsewhere in the paper.* The genome was submitted to SRA . Where can the functional annotation be accessed?

Level of Interest

Please indicate how interesting you found the manuscript: An article of importance in its field

Quality of Written English

Please indicate the quality of language in the manuscript: Needs some language corrections before being published

Declaration of Competing Interests

Please complete a declaration of competing interests, considering the following questions:

- Have you in the past five years received reimbursements, fees, funding, or salary from an organisation that may in any way gain or lose financially from the publication of this manuscript, either now or in the future?
- Do you hold any stocks or shares in an organisation that may in any way gain or lose financially from the publication of this manuscript, either now or in the future?
- Do you hold or are you currently applying for any patents relating to the content of the manuscript?
- Have you received reimbursements, fees, funding, or salary from an organization that holds or has applied for patents relating to the content of the manuscript?
- Do you have any other financial competing interests?
- Do you have any non-financial competing interests in relation to this paper?

If you can answer no to all of the above, write 'I declare that I have no competing interests' below. If your reply is yes to any, please give details below.

I declare that I have no competing interests

I agree to the open peer review policy of the journal. I understand that my name will be included on my report to the authors and, if the manuscript is accepted for publication, my named report including any attachments I upload will be posted on the website along with the authors' responses. I agree for my report to be made available under an Open Access Creative Commons CC-BY license (http://creativecommons.org/licenses/by/4.0/). I understand that any comments which I do not wish to be included in my named report can be included as confidential comments to the editors, which will not be published.

I agree to the open peer review policy of the journal
