# Supplementary material for: The draft genome assembly of Rhododendron delavayi Franch. var. delavayi
Source: Gigascience. 2017 Aug 26;6(10):1–11. doi: 10.1093/gigascience/gix076 (PMC5632301; doi:10.1093/gigascience/gix076)
Supplement: Reviewer-2-Report-(Revision-1).pdf [file gix076_Reviewer-2-Report-(Revision-1).pdf]

Reviewer Report

Title: The draft genome assembly of Rhododendron delavayi Franch. var. delavayi

Version: Revision 1 **Date:** 6/12/2017

Reviewer name: Naama Menda

Reviewer Comments to Author:

Lines 53-57 : please go over the paragraph and make it more clear - it is not clear if this paragraph is talking about the ease of hybridization or something with some subgenus taxa

Lines 72-73 : consider removing the words 'to facilitate future research' . The next sentence describes what is the expected outcome of the genome sequence

Table 6 - Does the column 'number' refer to number of genes?

Table 5 - the text points to table 4 (line 199) and then table 6 (line 204) , and no reference in the text to table 5

Gene annotation - please add number of unique genes in the species, that did not have any match to other known genes

Phylogenetic analysis - a figure with the trees would be helpful

I could not log into the authors' ftp site with the user and password stated in the response to reviewers

Level of Interest

Please indicate how interesting you found the manuscript: An article of importance in its field

Quality of Written English

Please indicate the quality of language in the manuscript: Acceptable

Declaration of Competing Interests

Please complete a declaration of competing interests, considering the following questions:

- Have you in the past five years received reimbursements, fees, funding, or salary from an organisation that may in any way gain or lose financially from the publication of this manuscript, either now or in the future?
- Do you hold any stocks or shares in an organisation that may in any way gain or lose financially from the publication of this manuscript, either now or in the future?
- Do you hold or are you currently applying for any patents relating to the content of the manuscript?
- Have you received reimbursements, fees, funding, or salary from an organization that holds or has applied for patents relating to the content of the manuscript?
- Do you have any other financial competing interests?
- Do you have any non-financial competing interests in relation to this paper?

If you can answer no to all of the above, write 'I declare that I have no competing interests' below. If your reply is yes to any, please give details below.

I declare that I have no competing interests

I agree to the open peer review policy of the journal. I understand that my name will be included on my report to the authors and, if the manuscript is accepted for publication, my named report including any attachments I upload will be posted on the website along with the authors' responses. I agree for my report to be made available under an Open Access Creative Commons CC-BY license (http://creativecommons.org/licenses/by/4.0/). I understand that any comments which I do not wish to be included in my named report can be included as confidential comments to the editors, which will not be published.

I agree to the open peer review policy of the journal
