# Supplementary material for: The draft genome assembly of Rhododendron delavayi Franch. var. delavayi
Source: Gigascience. 2017 Aug 26;6(10):1–11. doi: 10.1093/gigascience/gix076 (PMC5632301; doi:10.1093/gigascience/gix076)
Supplement: Reviewer-3-Report-(Original-Submission).pdf [file gix076_Reviewer-3-Report-(Original-Submission).pdf]

Reviewer Report

Title: The draft genome assembly of Rhododendron delavayi Franch. var. delavayi

Version: Original Submission **Date:** 3/27/2017

Reviewer name: John Hamilton

Reviewer Comments to Author:

The manuscript describes the sequencing and assembly of the ornamental plant Rhododendron delavayi. The manuscript was submitted as a "Data Note" and the manuscript is focused appropriately on the generation of the assembly, genome annotation and data access.Overall the manuscript adequately describes the sequencing, assembly, annotation for R. delavayi.The manuscript has several passages (mostly in the Introduction) where the phrasing is odd or there are grammar mistakes and it should be revised before being published.Minor Comments:* The versions for all the software used should be stated.* Line 45 - high-through-put sequencing -> genome sequencing* Line 49 - research on bioinformatics -> genomics (or functional genomics)* Line 55 - morphology -> appearance* Lines 74-77 - odd phrasing to describe the mate-pair library protocol* Line 100 - Ma Ying azalea - single use of this name in the manuscript. It's not consistent and potentially confusing.* Line 120 -"proper parameters were chosen"* Line 171 - typo - Solanum tuberosum* Line 196 - typo - GLEAN* Table 6 - Numbers column missing the final digit

Level of Interest

Please indicate how interesting you found the manuscript: An article of limited interest

Quality of Written English

Please indicate the quality of language in the manuscript: Needs some language corrections before being published

Declaration of Competing Interests

Please complete a declaration of competing interests, considering the following questions:

- Have you in the past five years received reimbursements, fees, funding, or salary from an organisation that may in any way gain or lose financially from the publication of this manuscript, either now or in the future?
- Do you hold any stocks or shares in an organisation that may in any way gain or lose financially from the publication of this manuscript, either now or in the future?
- Do you hold or are you currently applying for any patents relating to the content of the manuscript?

- Have you received reimbursements, fees, funding, or salary from an organization that holds or has applied for patents relating to the content of the manuscript?
- Do you have any other financial competing interests?
- Do you have any non-financial competing interests in relation to this paper?

If you can answer no to all of the above, write 'I declare that I have no competing interests' below. If your reply is yes to any, please give details below.

I declare that I have no competing interests

I agree to the open peer review policy of the journal. I understand that my name will be included on my report to the authors and, if the manuscript is accepted for publication, my named report including any attachments I upload will be posted on the website along with the authors' responses. I agree for my report to be made available under an Open Access Creative Commons CC-BY license (http://creativecommons.org/licenses/by/4.0/). I understand that any comments which I do not wish to be included in my named report can be included as confidential comments to the editors, which will not be published.

I agree to the open peer review policy of the journal
